# Supplementary material for: Design, Synthesis, Herbicidal Activity, and Structure–Activity Relationship Study of Novel 6-(5-Aryl-Substituted-1-Pyrazolyl)-2-Picolinic Acid as Potential Herbicides
Source: Molecules. 2023 Feb 2;28(3):1431. doi: 10.3390/molecules28031431 (PMC9920234; doi:10.3390/molecules28031431)
Supplement: Supplementary file 1 [file molecules-28-01431-s001.zip › molecules-2155307-supplementary.pdf]

Supplementary Materials:

Design, Synthesis, Herbicidal Activity, and Structure-activity Relationship Study of Novel 6-(5-Aryl-substituted-1-pyrazolyl)-2-picolinic Acid as Potential Herbicides

Tong Feng [1,2], Qing Liu [1,2], Zhi-Yuan Xu [1,2], Hui-Ting Li [1], Wei Wei [1], Rong-Chuan Shi [1], Li Zhang [1,2], Yi-Ming Cao [1,2*] and Shang-Zhong Liu [1,2*].

[1] Innovation Center of Pesticide Research, Department of Applied Chemistry, College of Science, China Agricultural University, Beijing 100193, China;

[2] Key Laboratory of National Forestry and Grassland Administration on Pest Chemical Control, China Agricultural Univrsity, Beijing 100193, China

* Correspondence: caoym@cau.edu.cn (Y.-M.C.); shangzho@cau.edu.cn (S.-Z.L.); Tel.: +86-10-62731070 (S.-Z.L.).

The NMR spectrogram of title compounds

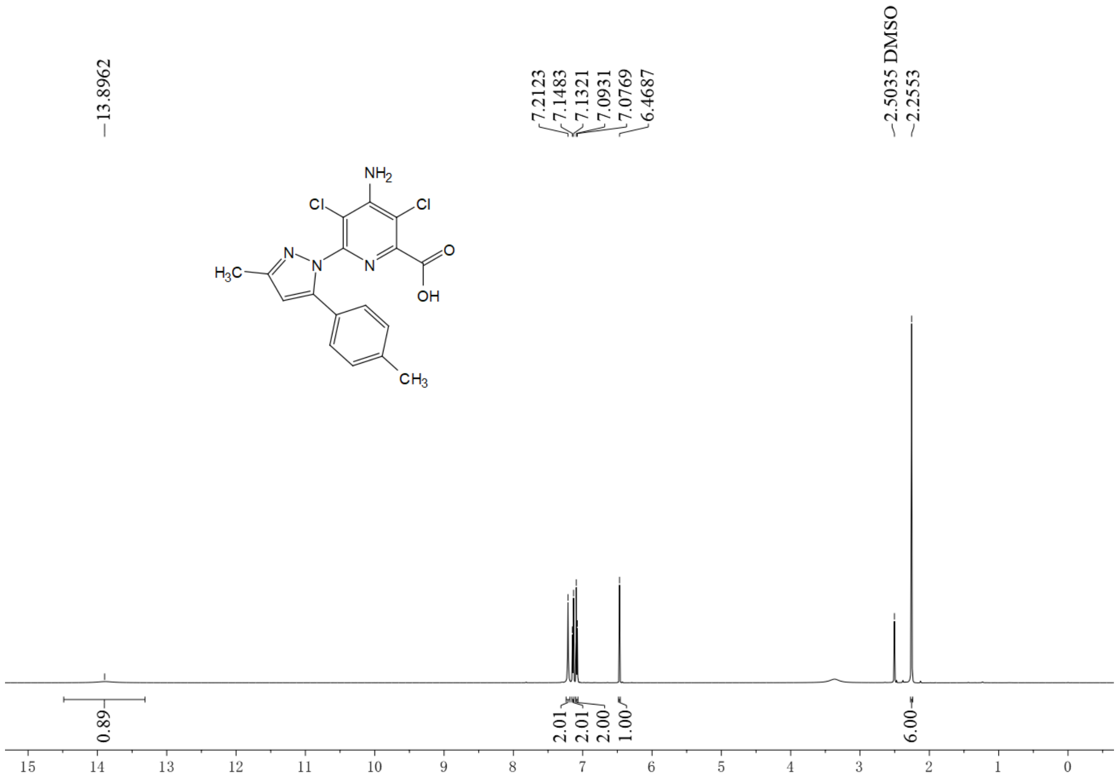

Figure S1. ^{1}H NMR spectrum of compound V-1.

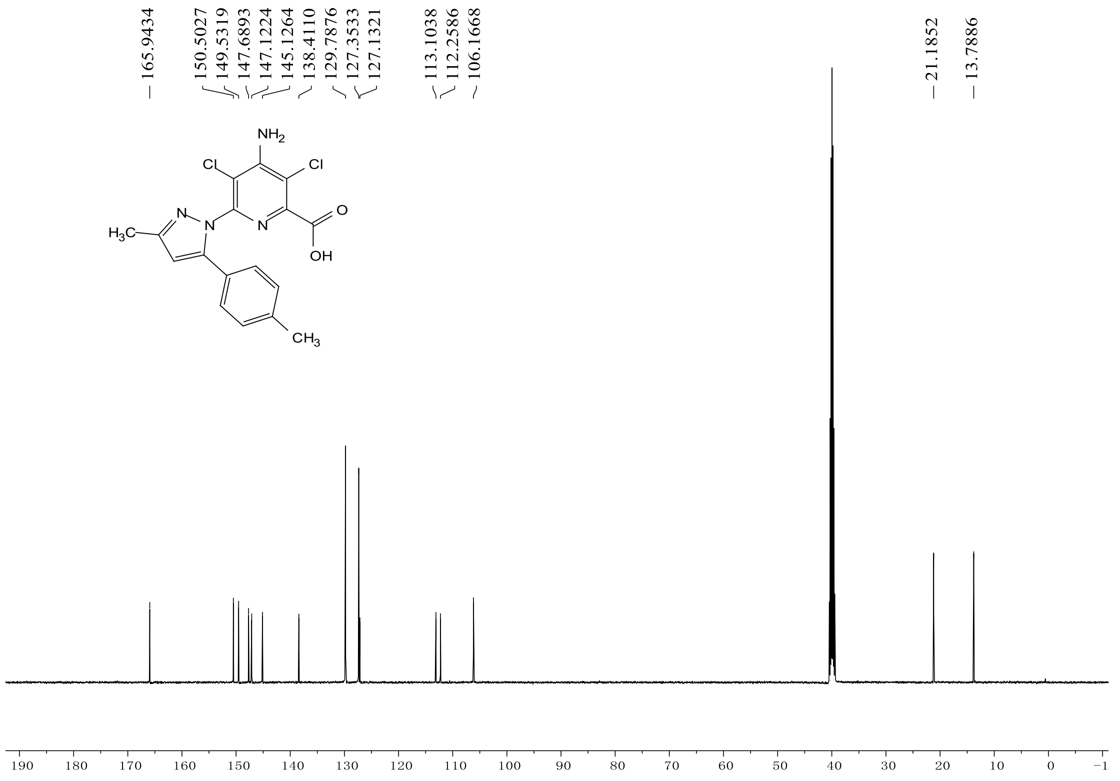

Figure S2. ^{13}C NMR spectrum of compound V-1.

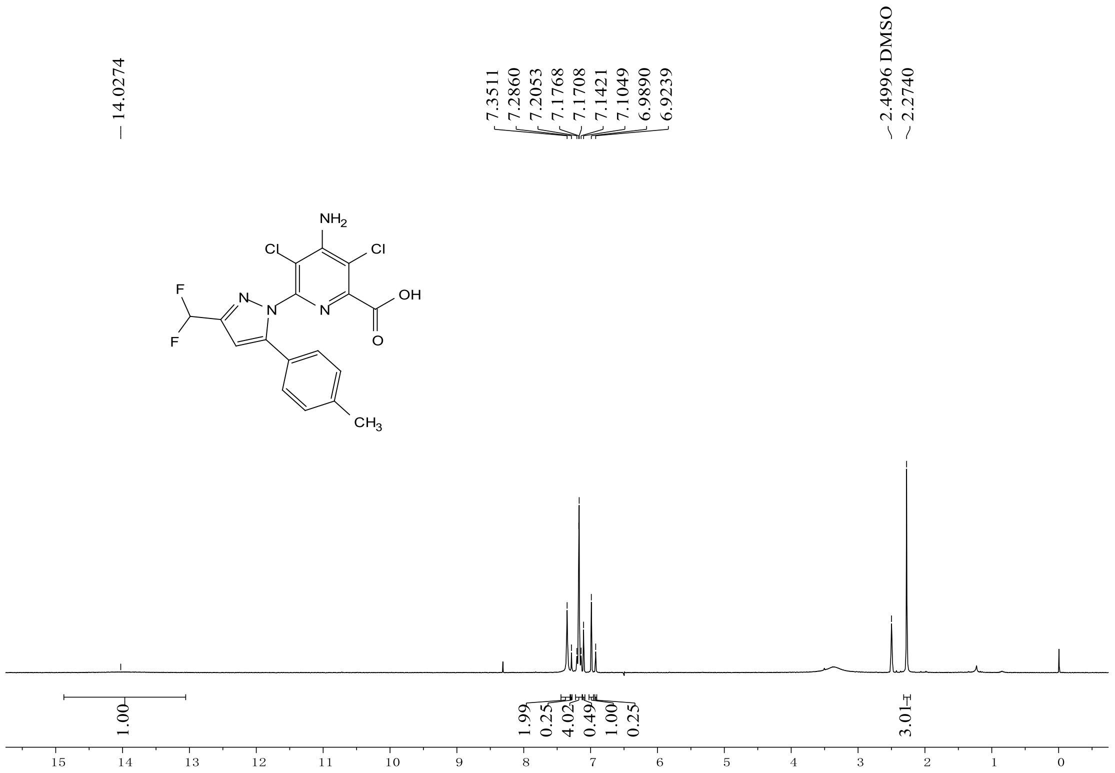

Figure S3. ^{1}H NMR spectrum of compound V-2.

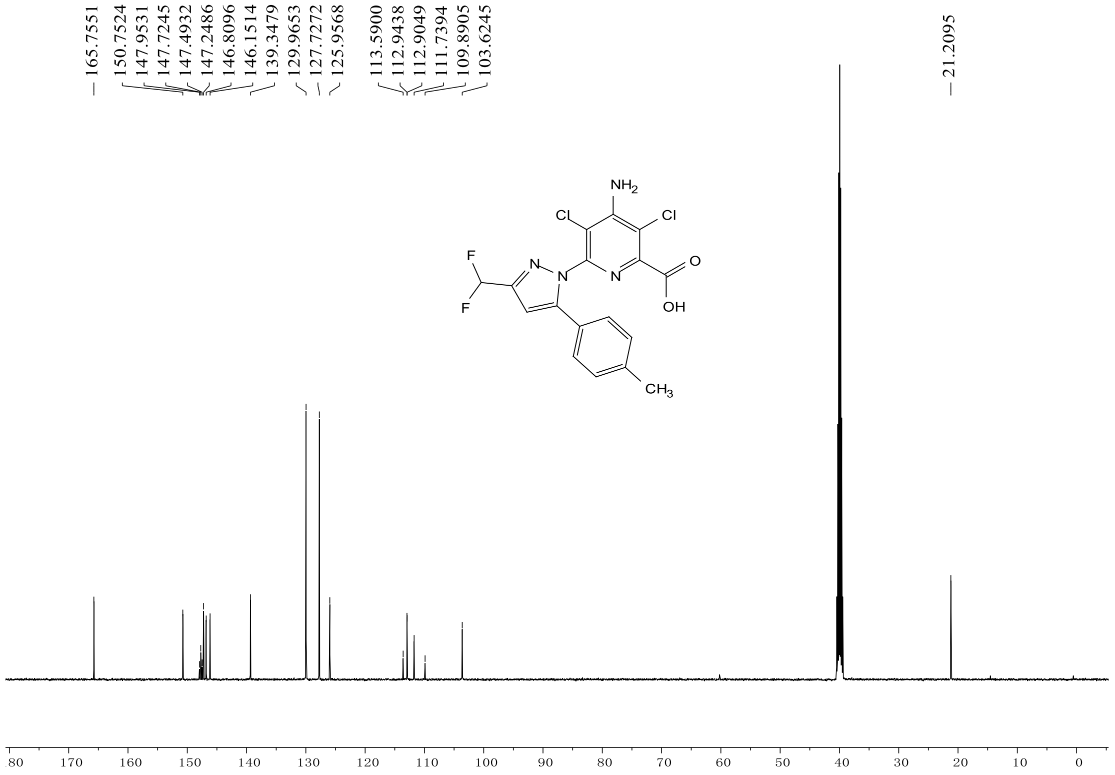

Figure S4. ^{13}C NMR spectrum of compound V-2.

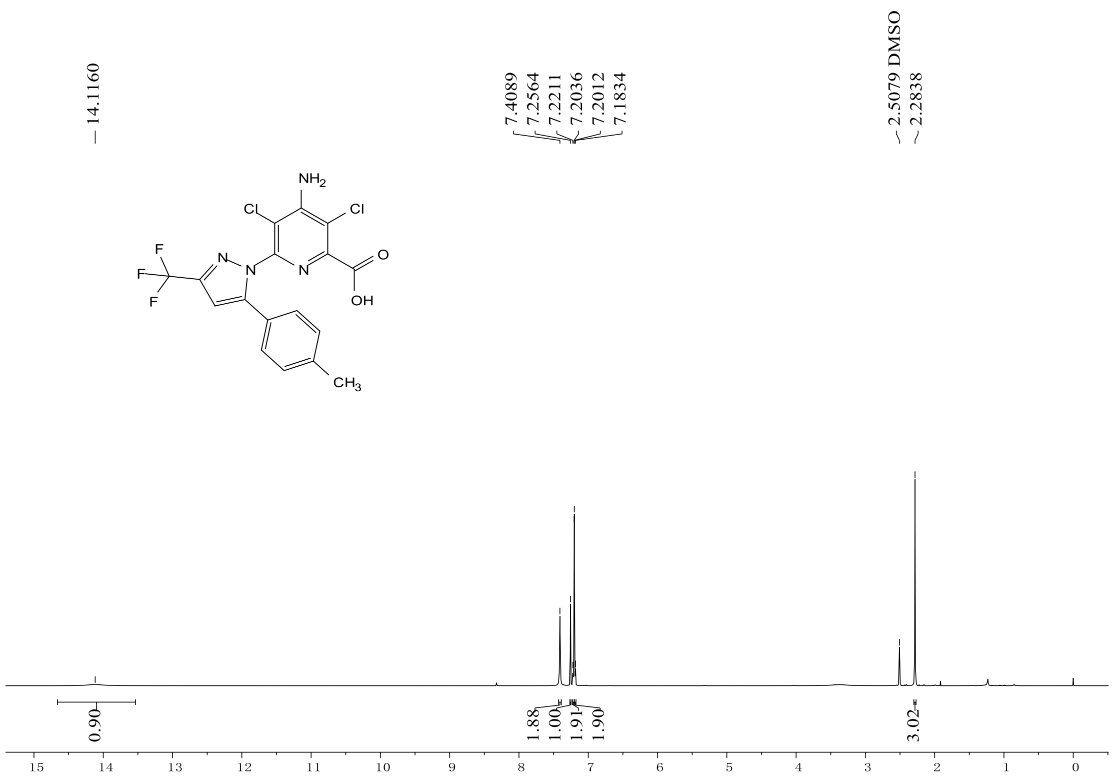

Figure S5. ^{1}H NMR spectrum of compound V-3.

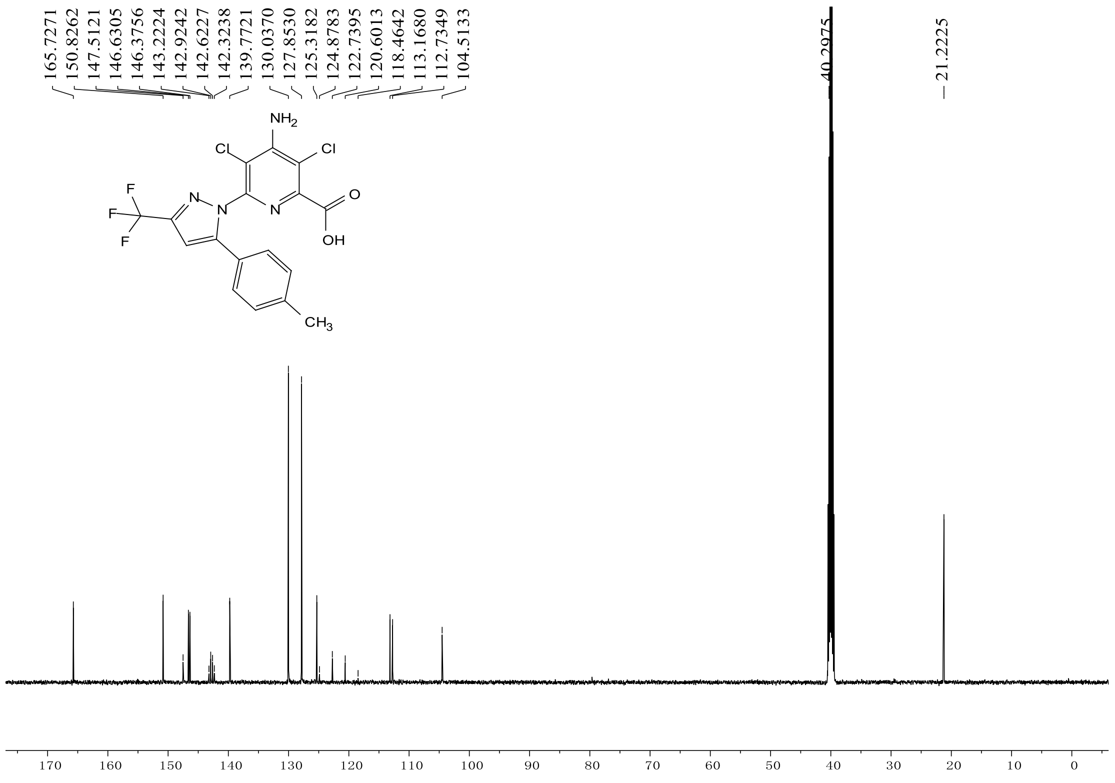

Figure S6. ^{13}C NMR spectrum of compound V-3.

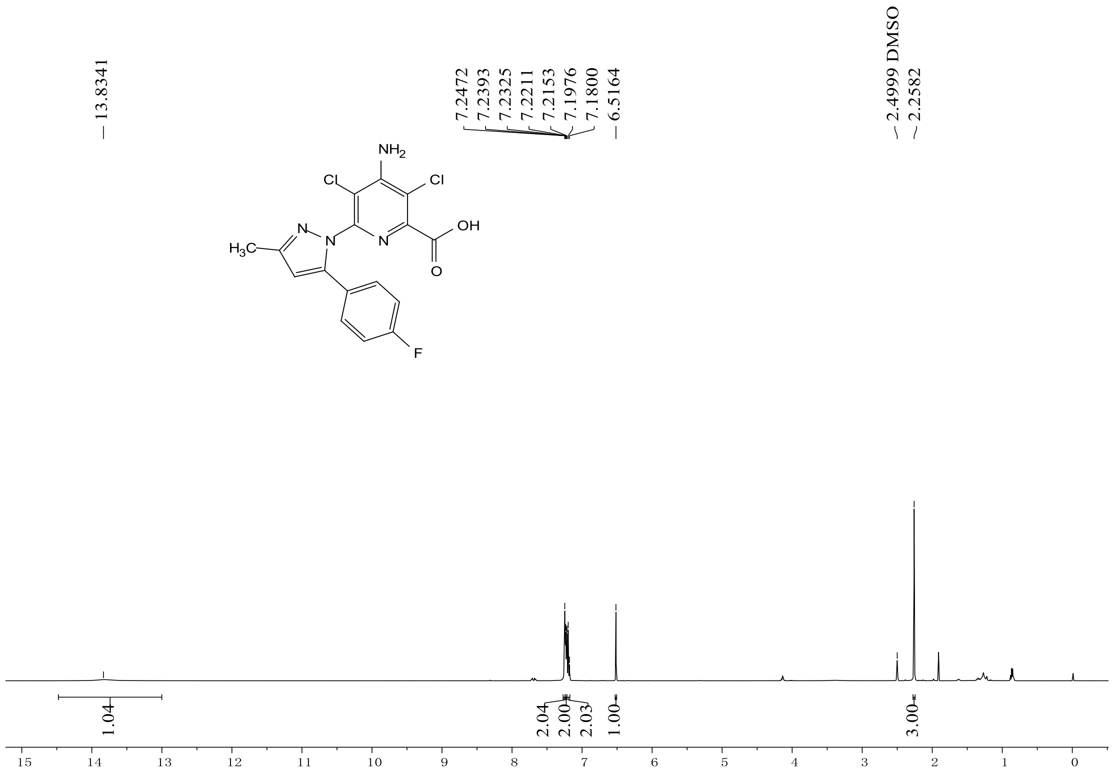

Figure S7. ^{1}H NMR spectrum of compound V-4.

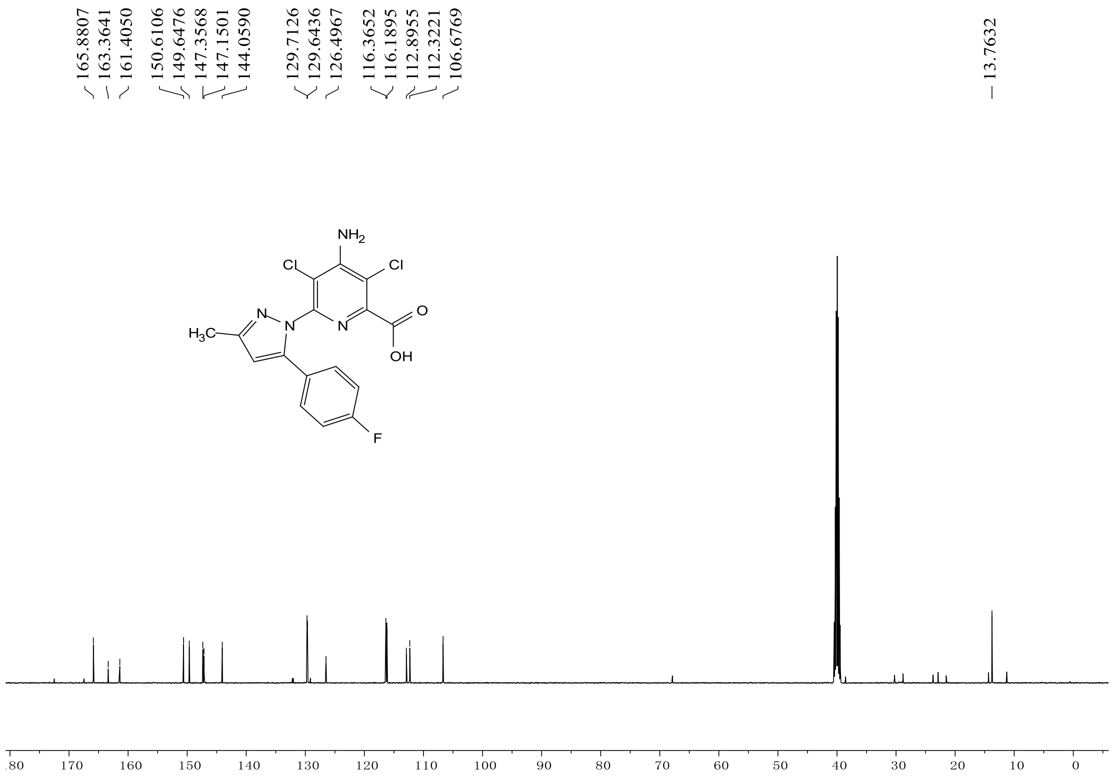

Figure S8. ^{13}C NMR spectrum of compound V-4.

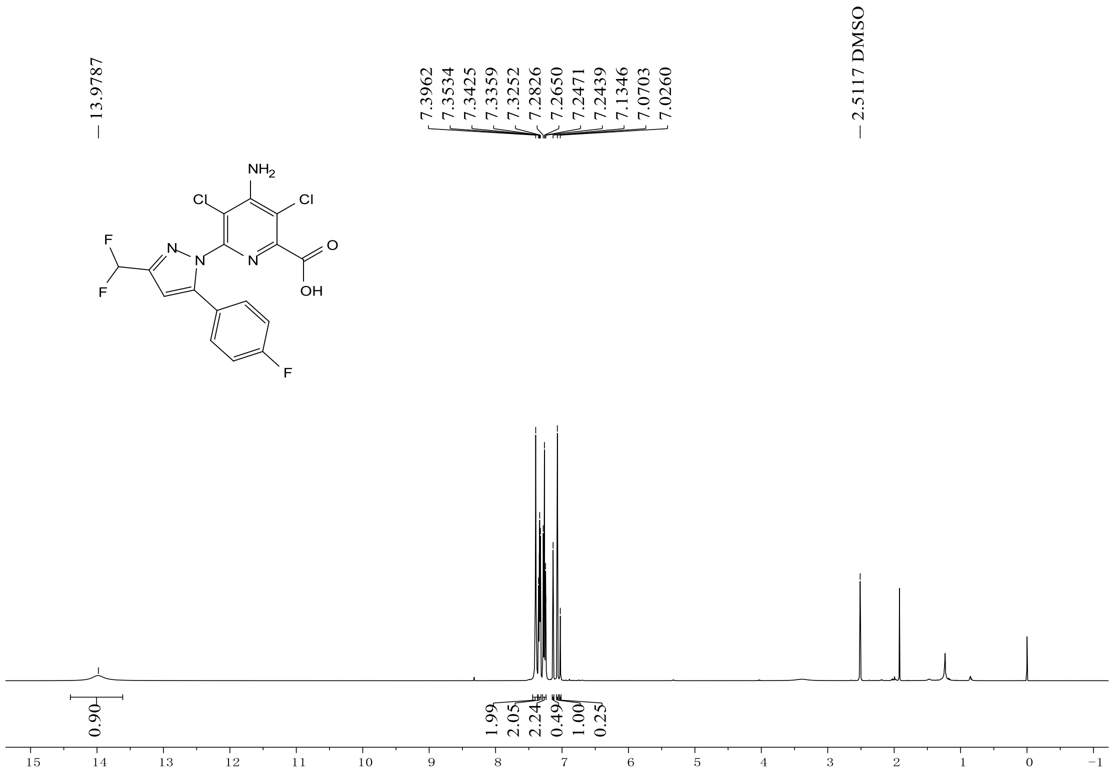

Figure S9. ^{1}H NMR spectrum of compound V-5.

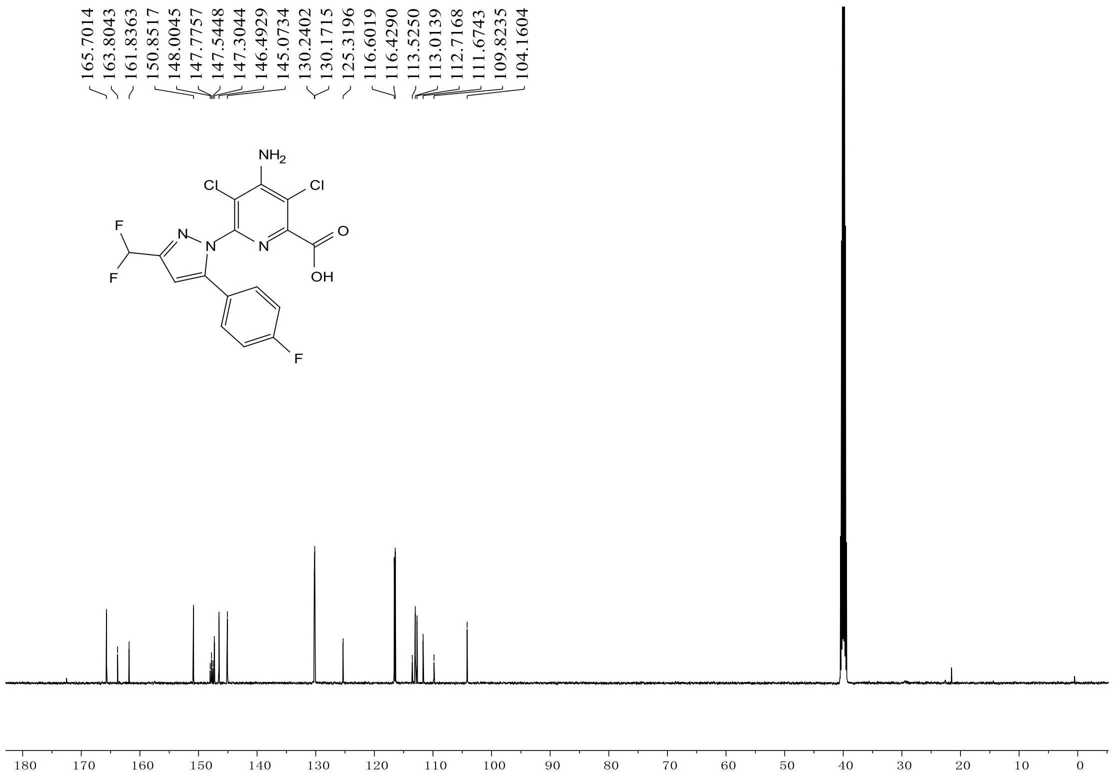

Figure S10. ^{13}C NMR spectrum of compound V-5.

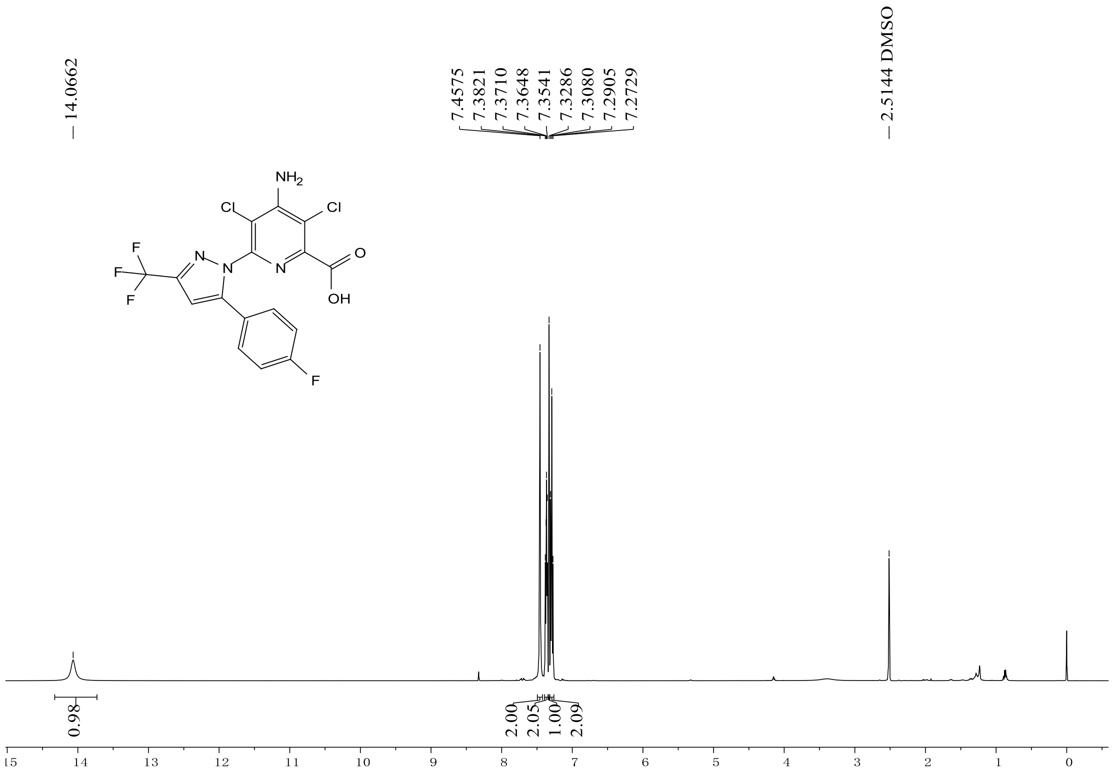

Figure S11. ^{1}H NMR spectrum of compound V-6.

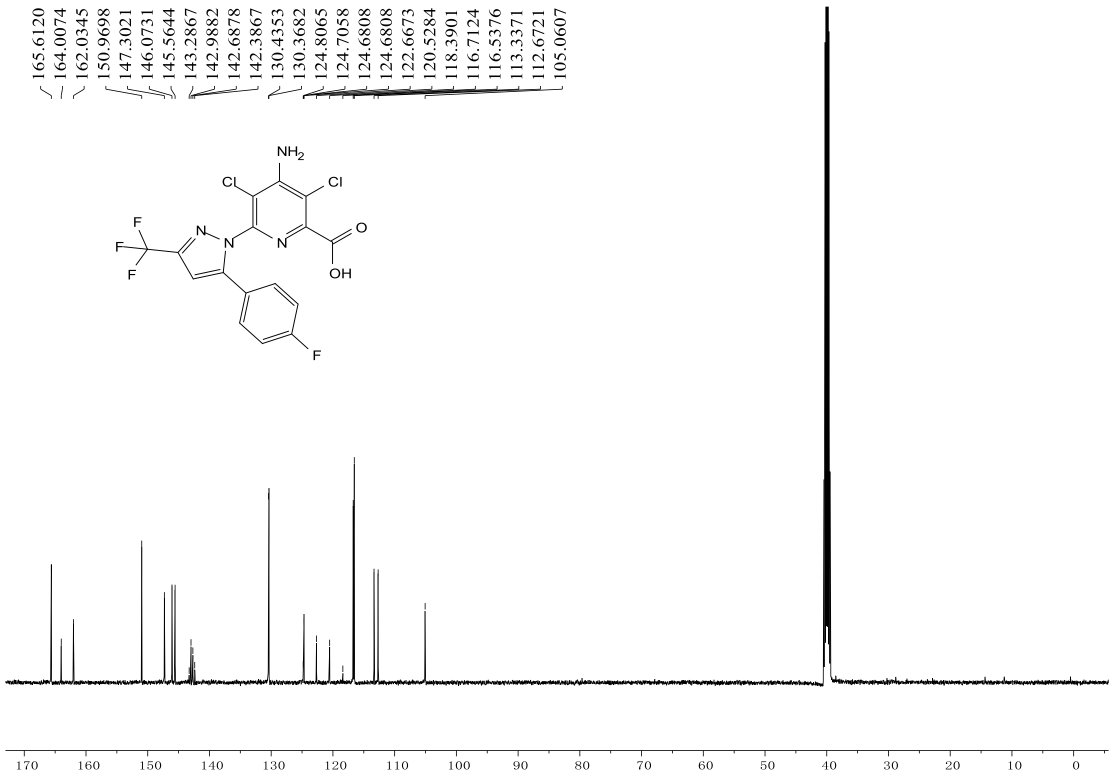

Figure S12. ^{13}C NMR spectrum of compound V-6.

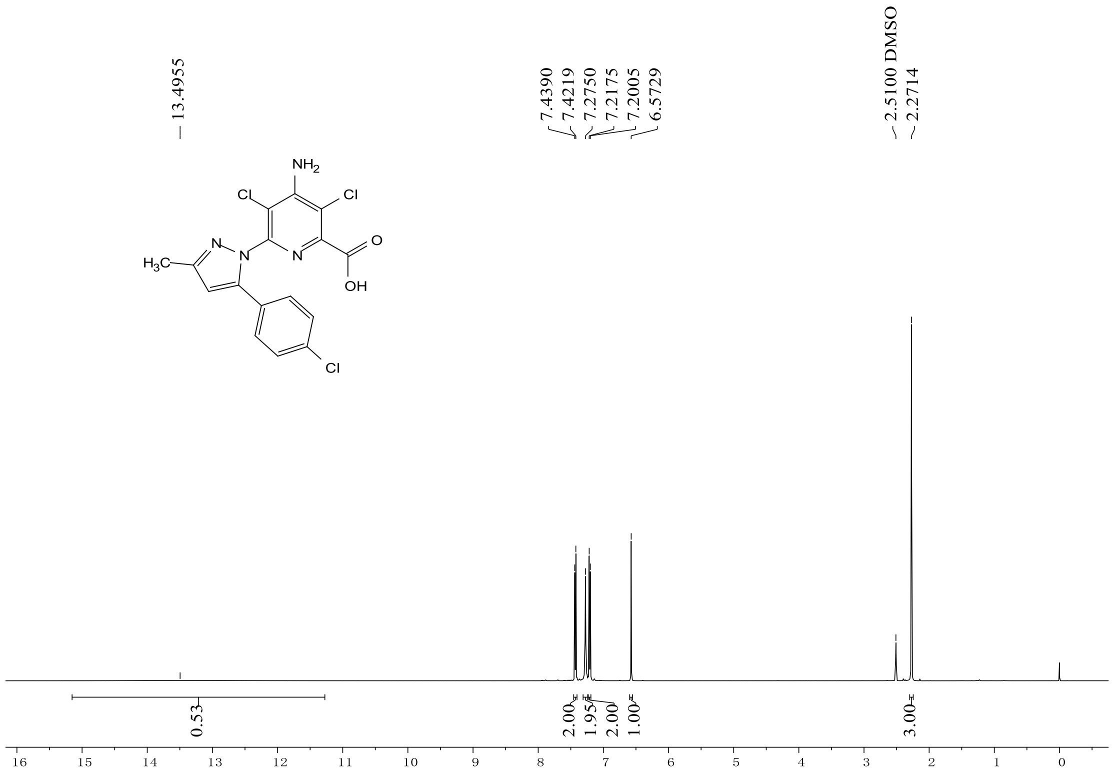

Figure S13. ^{1}H NMR spectrum of compound V-7.

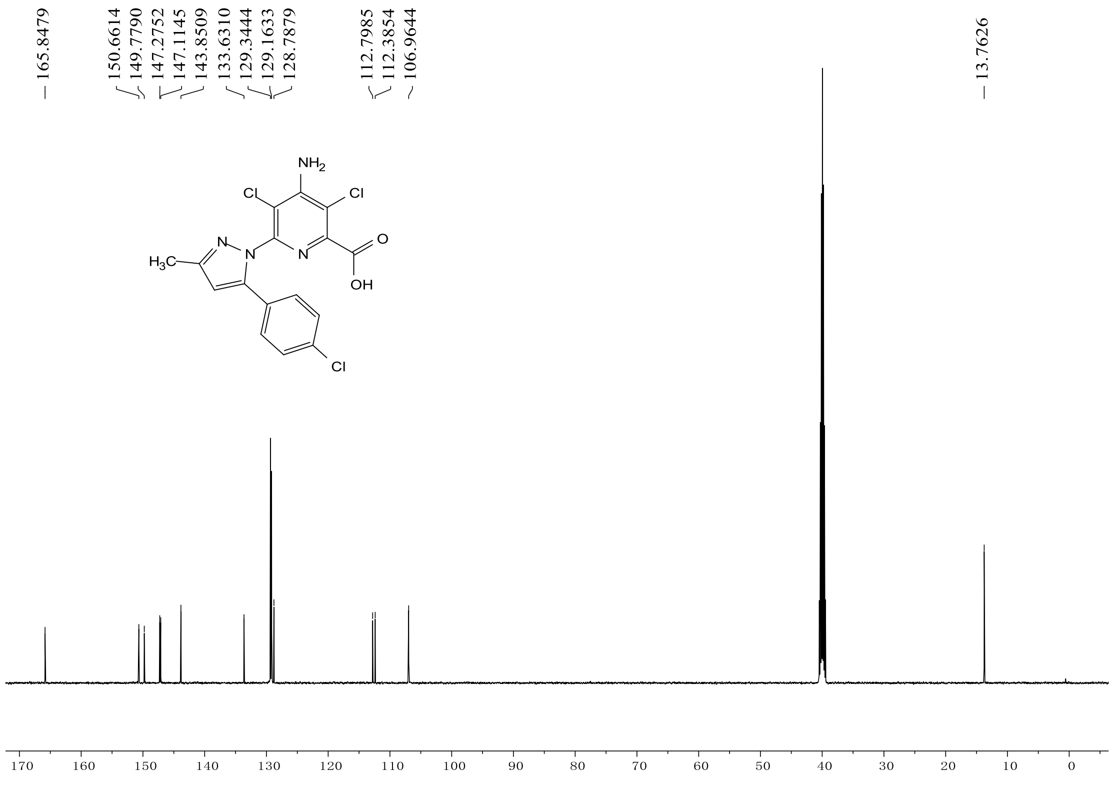

Figure S14. ^{13}C NMR spectrum of compound V-7.

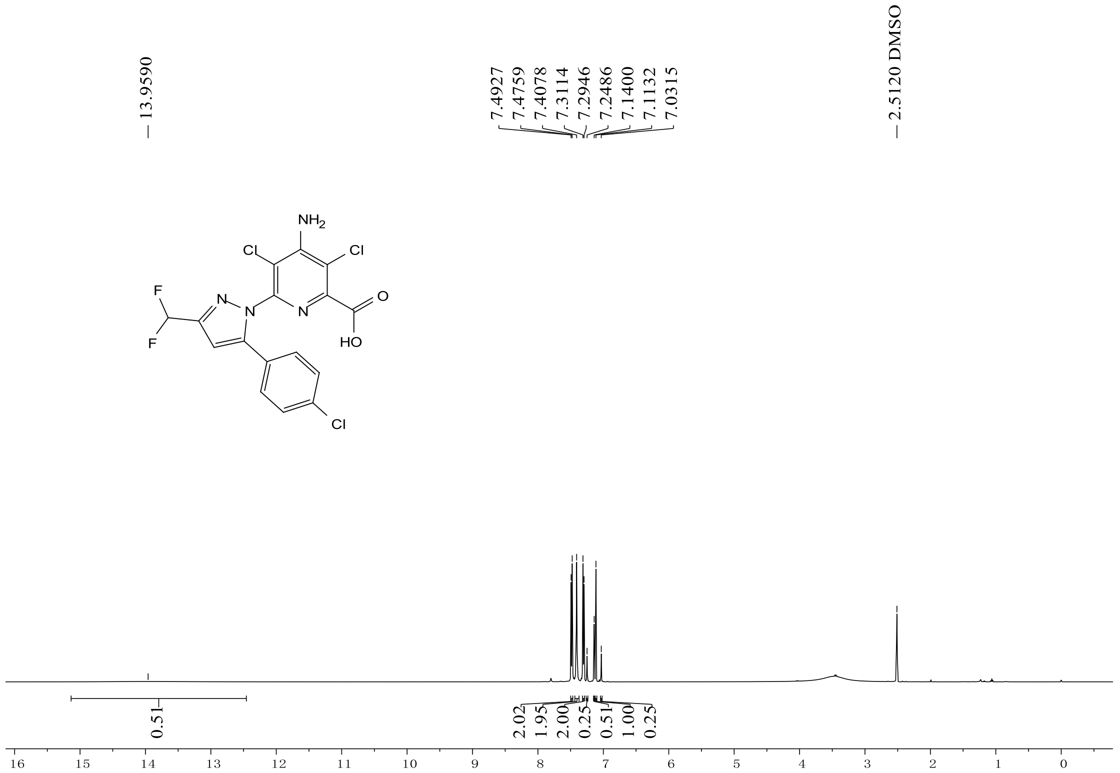

Figure S15. ^{1}H NMR spectrum of compound V-8.

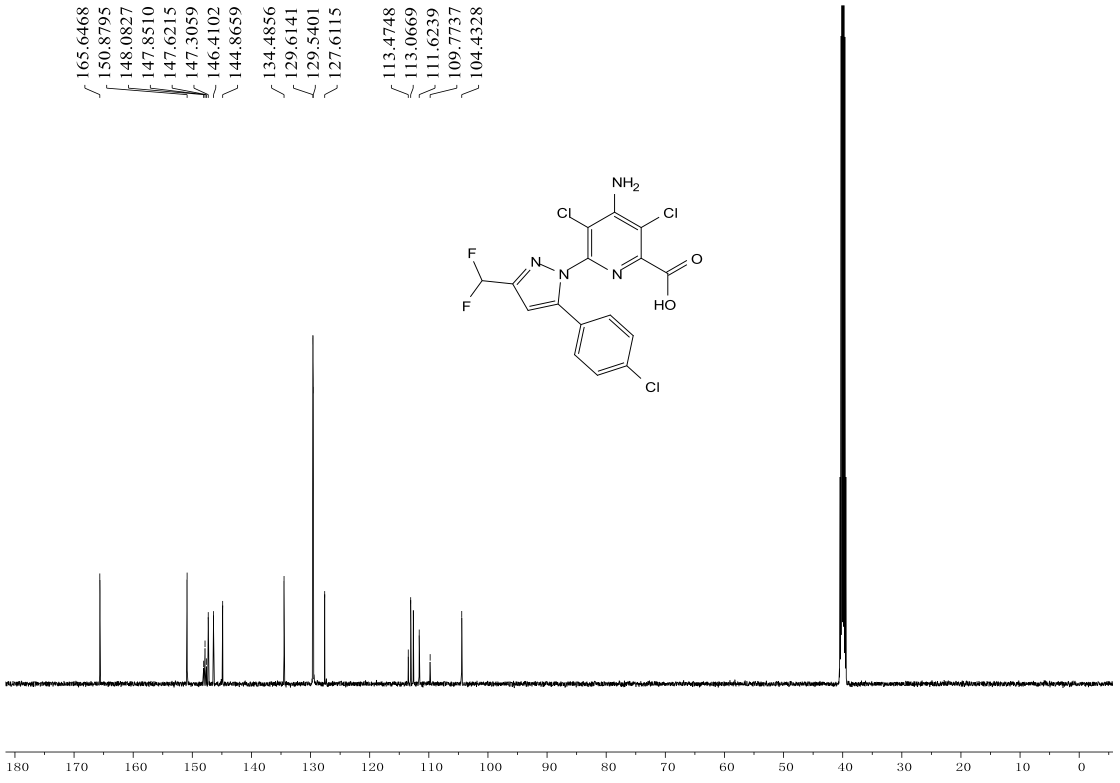

Figure S16. ^{13}C NMR spectrum of compound V-8.

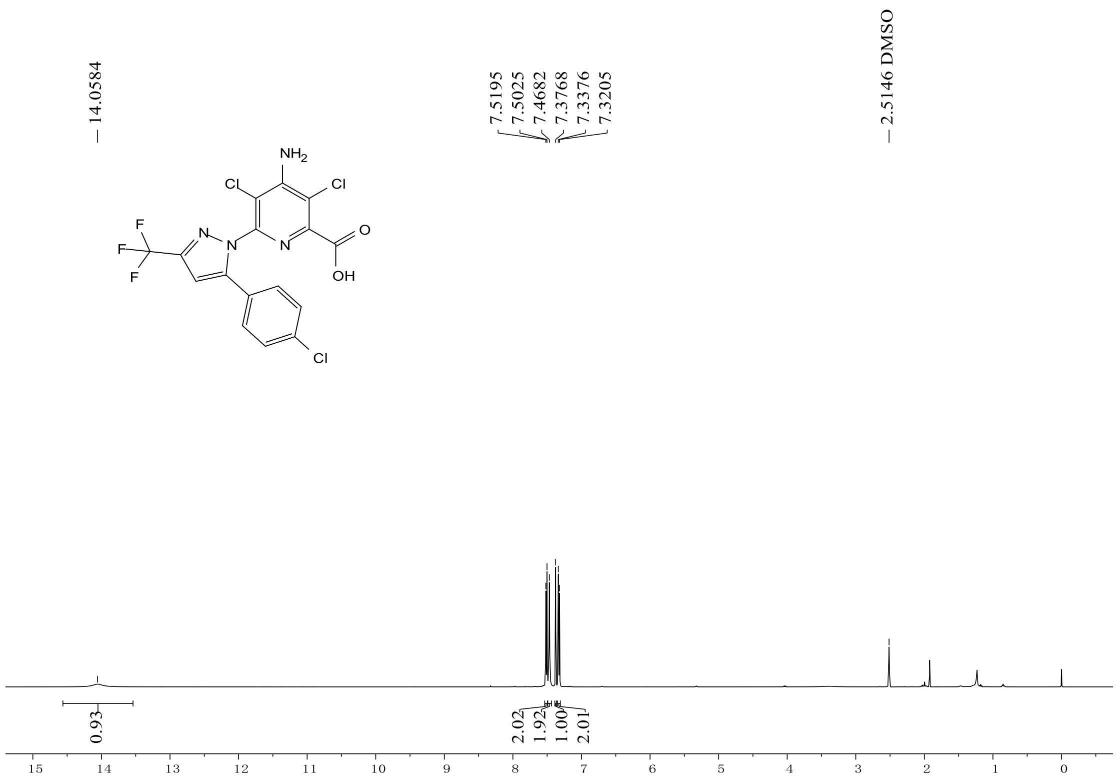

Figure S17. ^{1}H NMR spectrum of compound V-9.

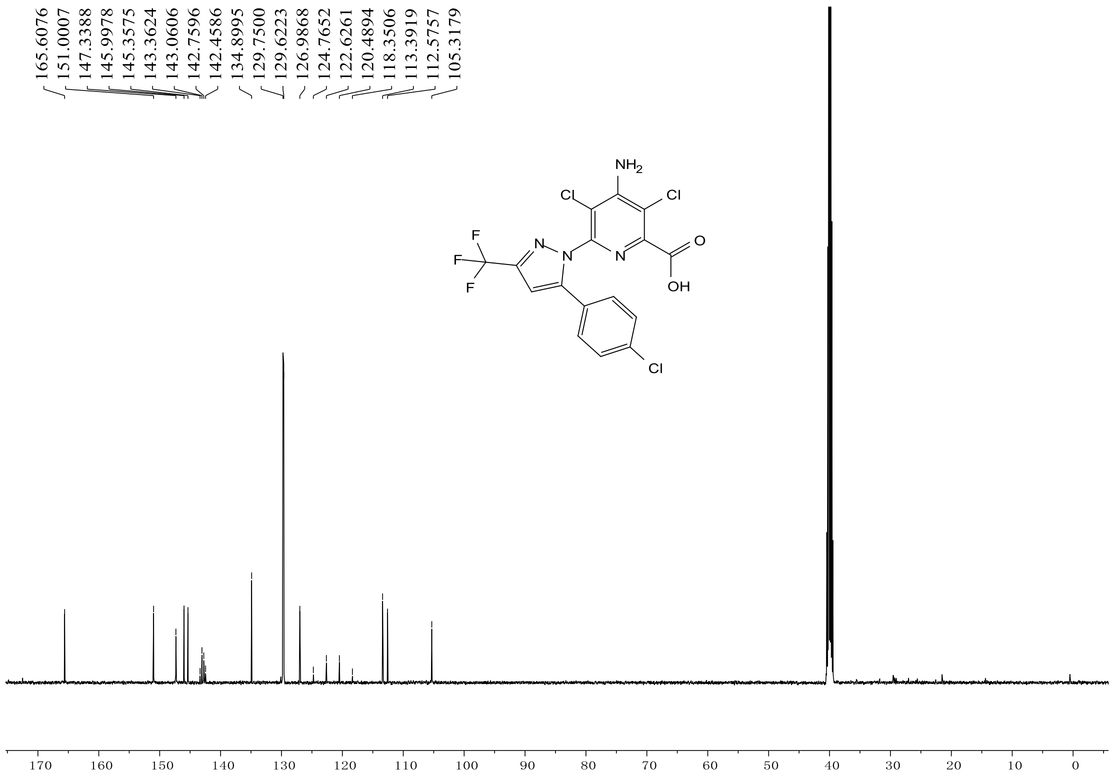

Figure S18. ^{13}C NMR spectrum of compound V-9.

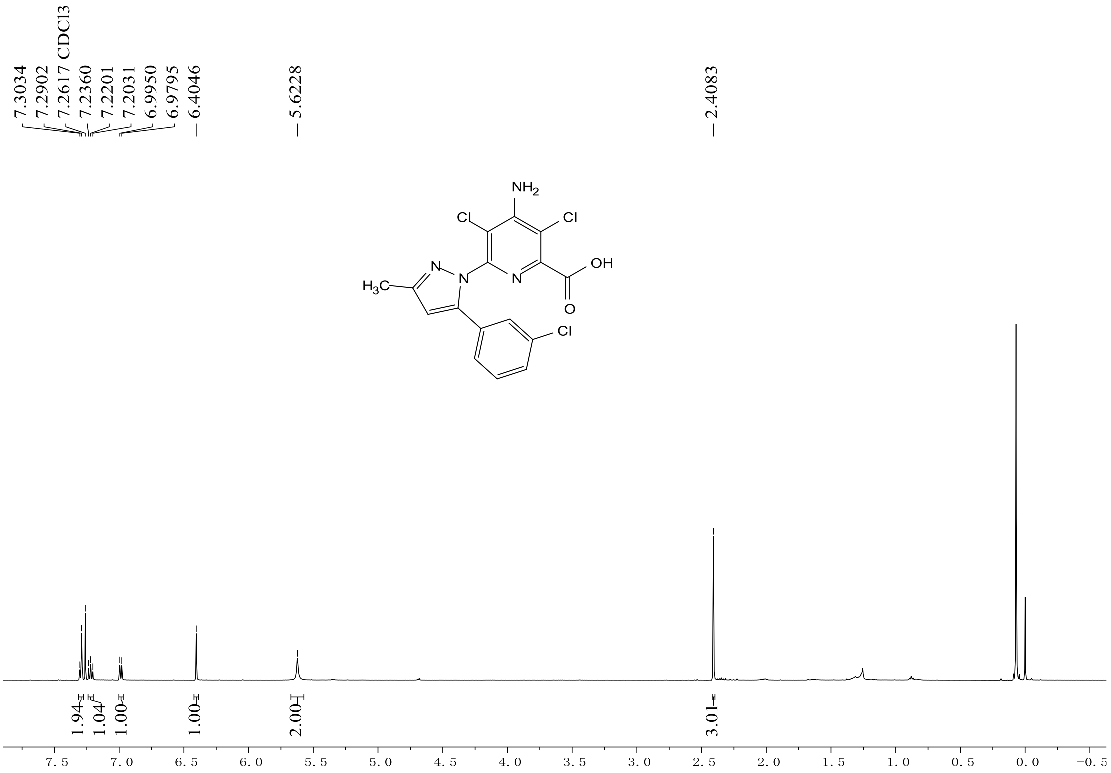

Figure S19. ^{1}H NMR spectrum of compound V-10.

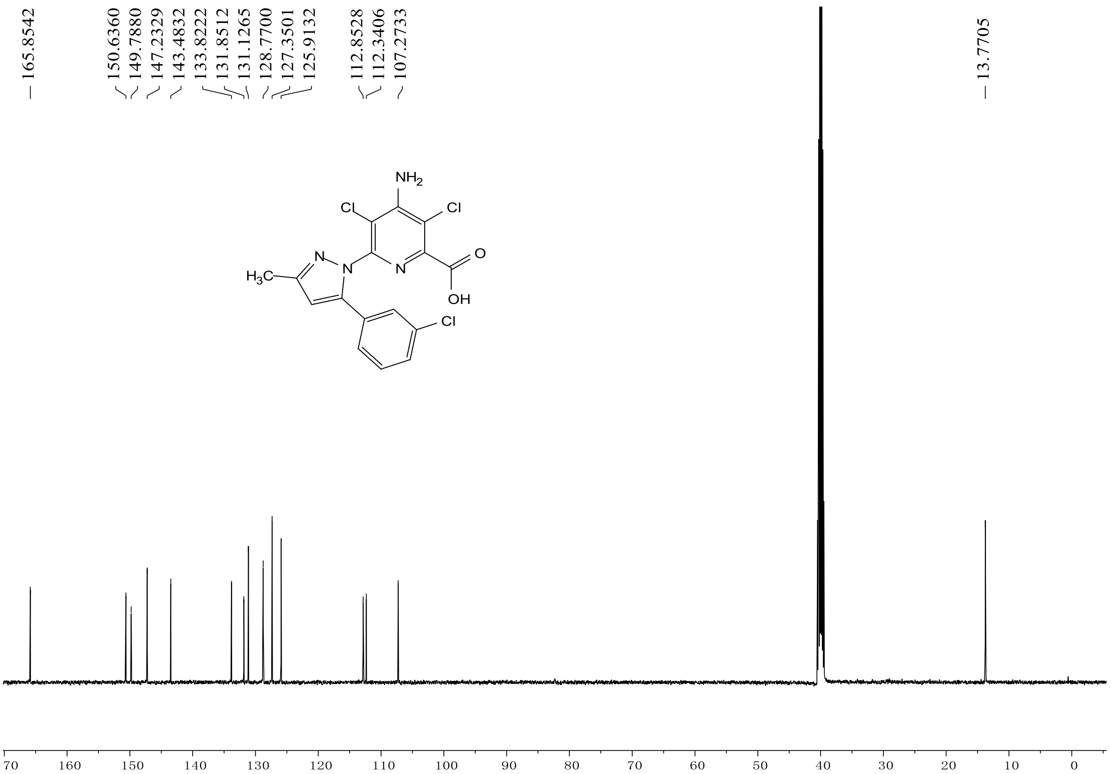

Figure S20. ^{13}C NMR spectrum of compound V-10.

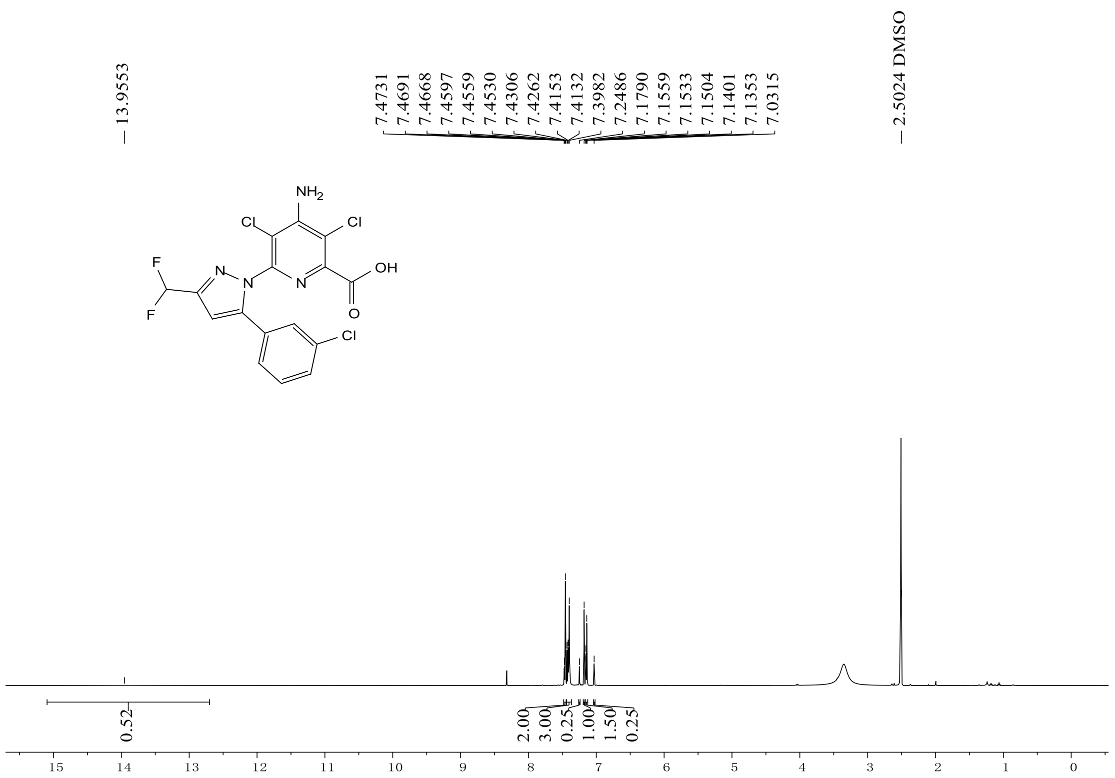

Figure S21. ^{1}H NMR spectrum of compound V-11.

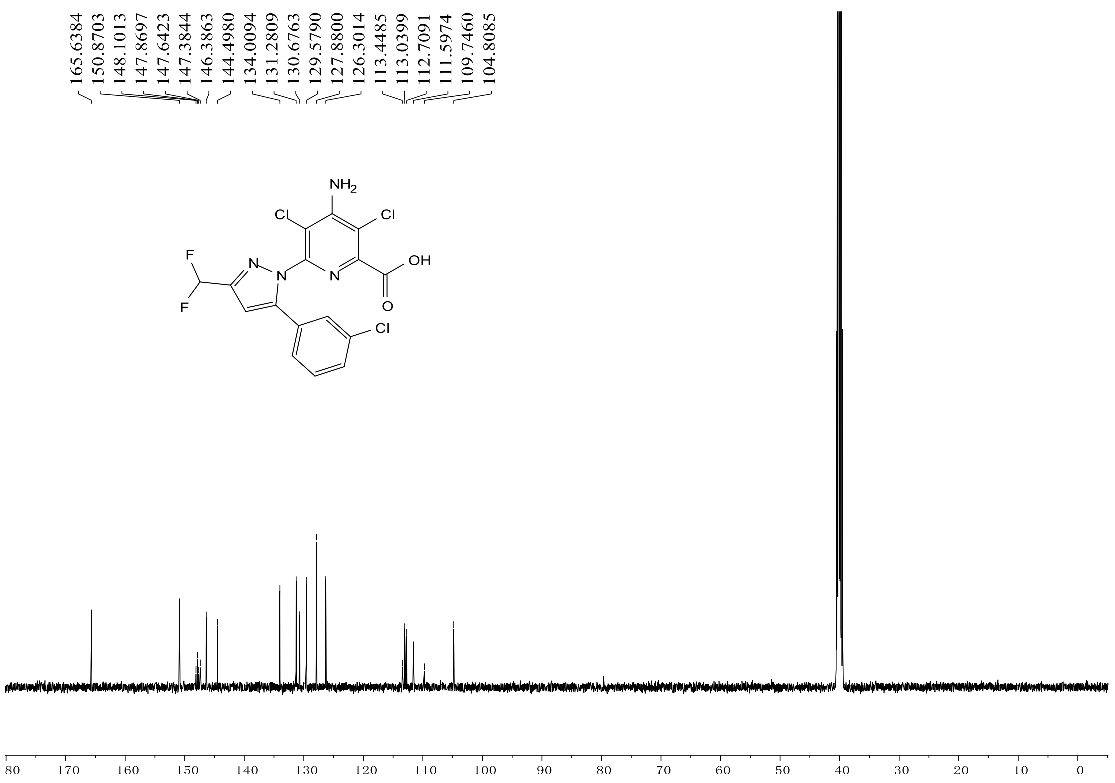

Figure S22. ^{13}C NMR spectrum of compound V-11.

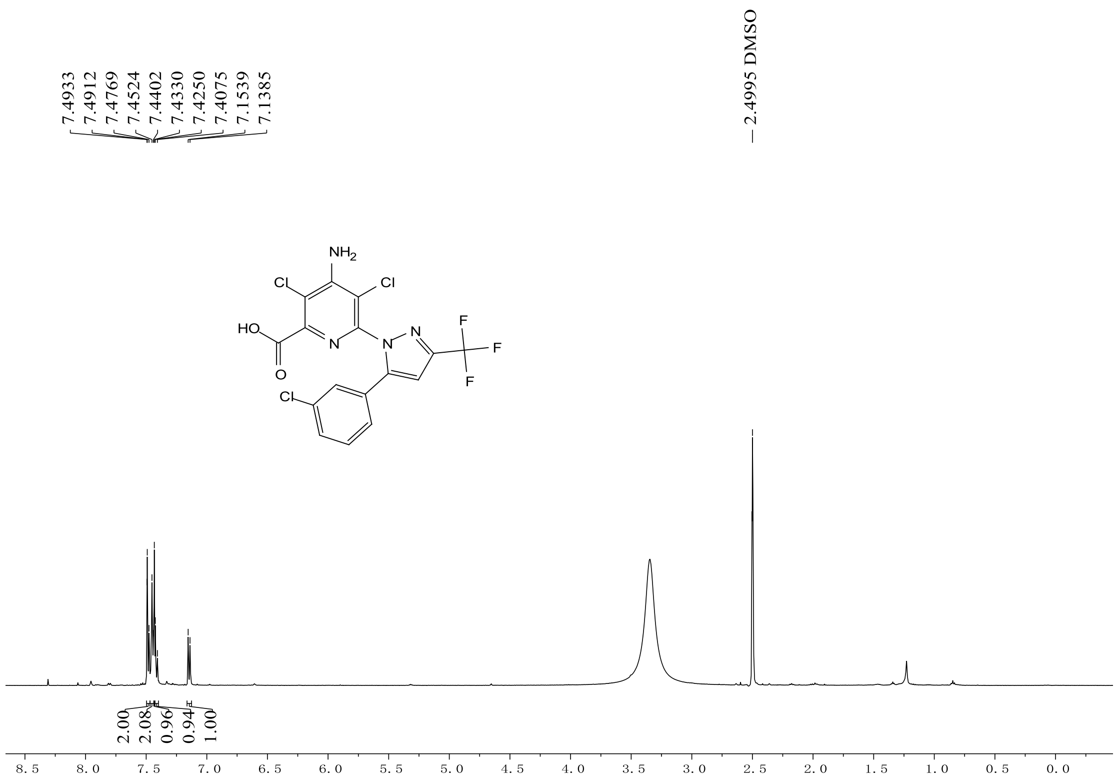

Figure S23. ^{1}H NMR spectrum of compound V-12.

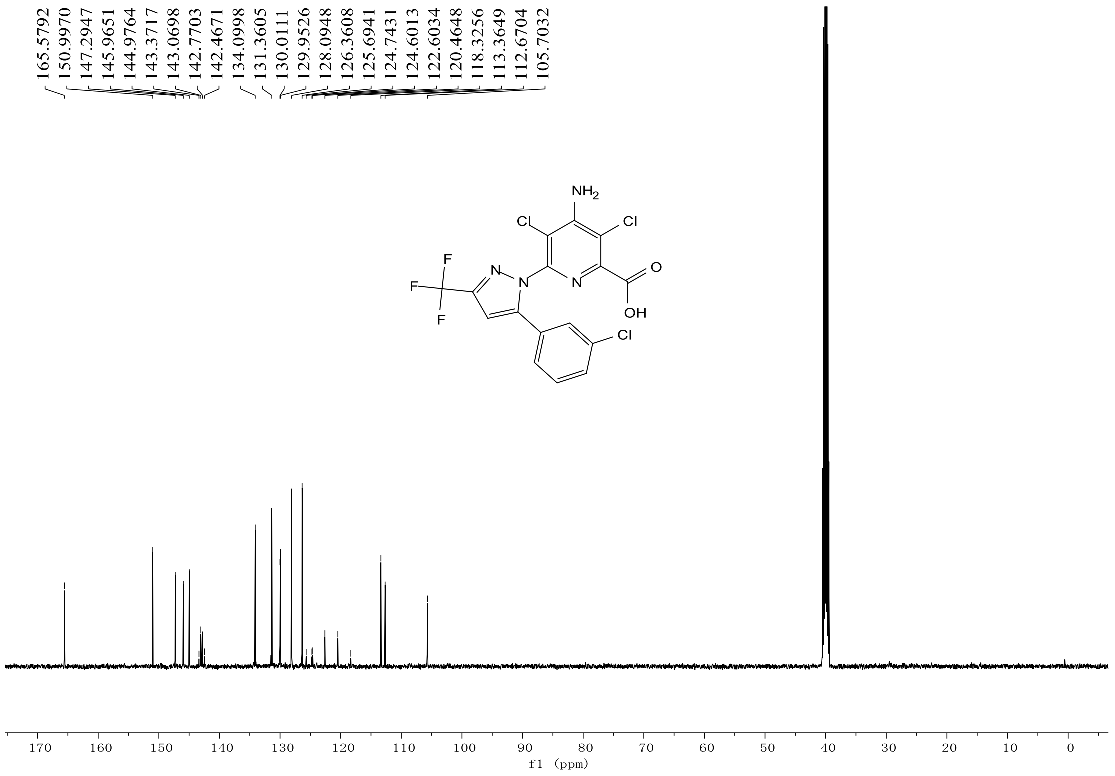

Figure S24. ^{13}C NMR spectrum of compound V-12.

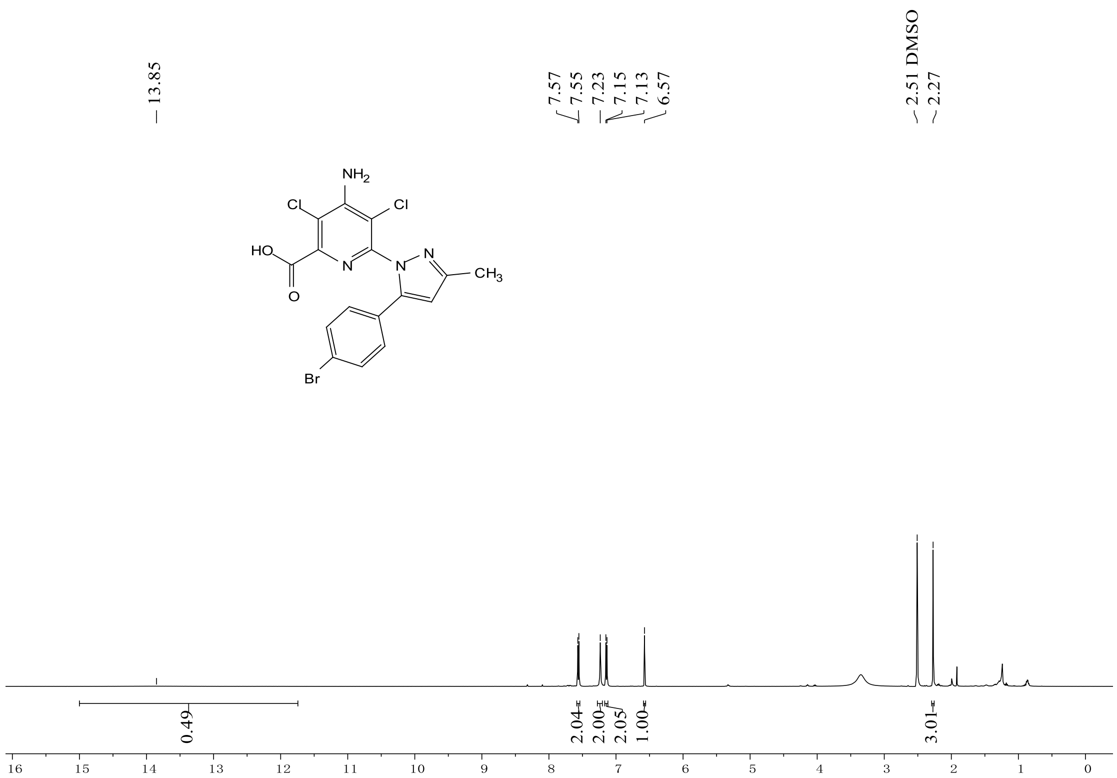

Figure S25. ^{1}H NMR spectrum of compound V-13.

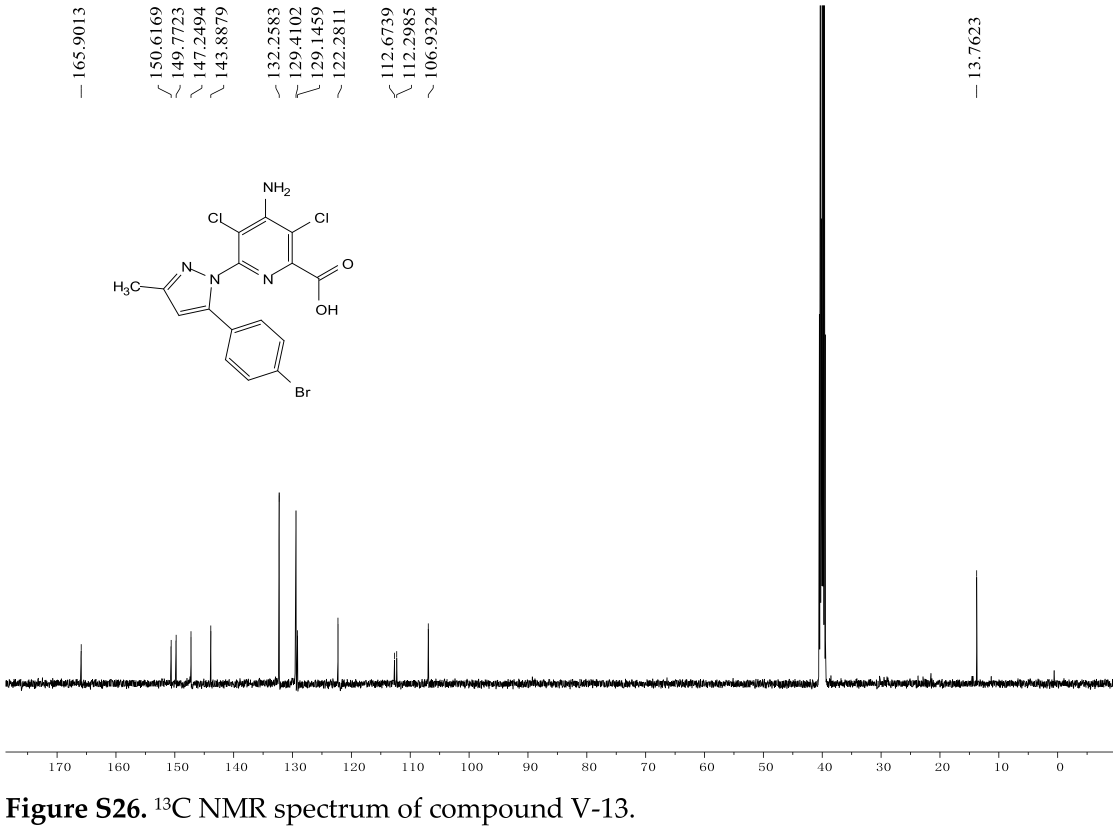

Figure S26. ^{13}C NMR spectrum of compound V-13.

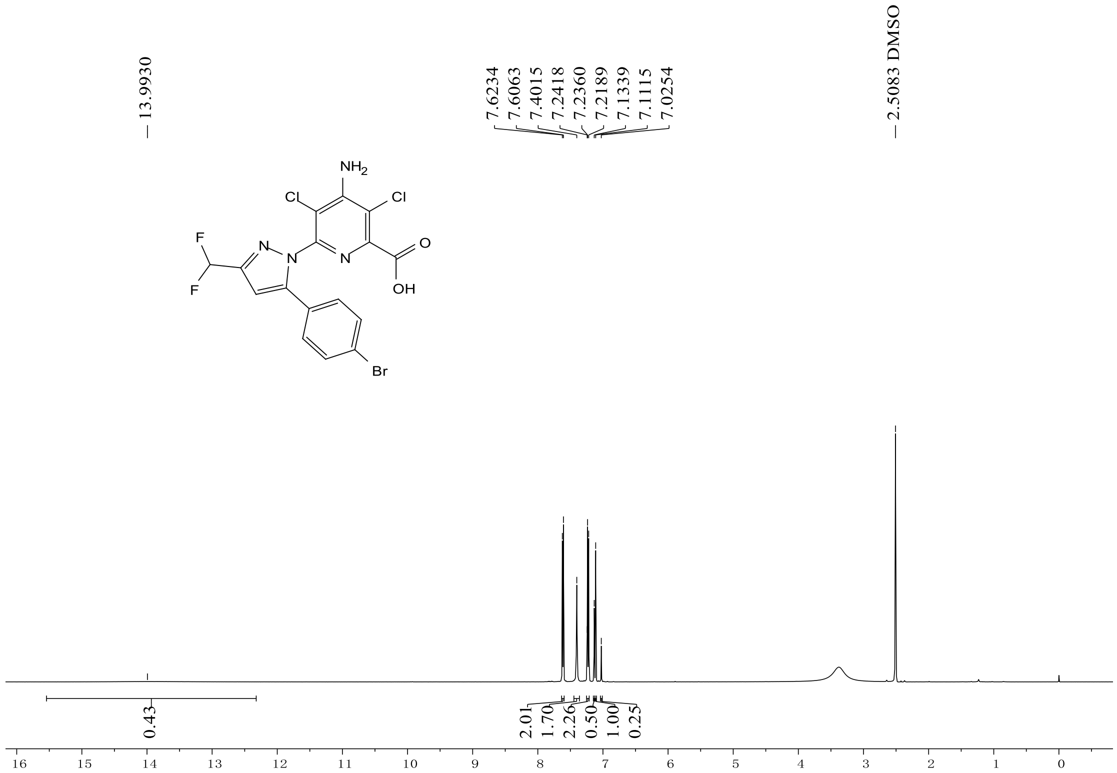

Figure S27. ^{1}H NMR spectrum of compound V-14.

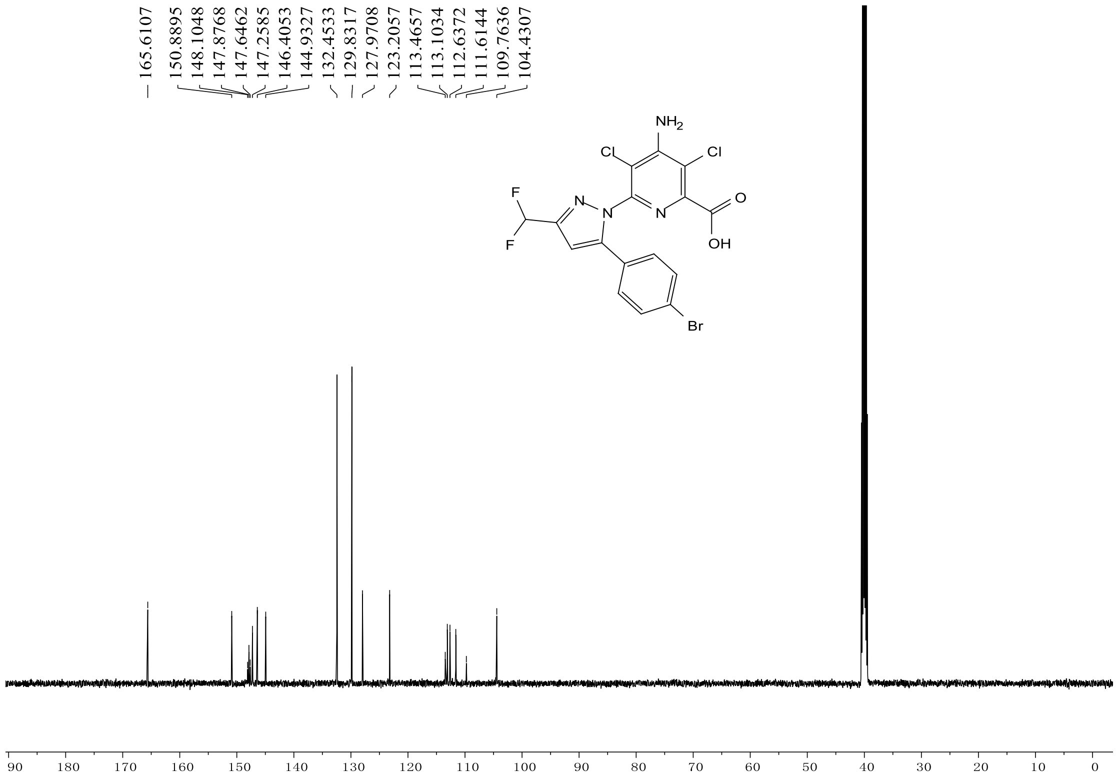

Figure S28. ^{13}C NMR spectrum of compound V-14.

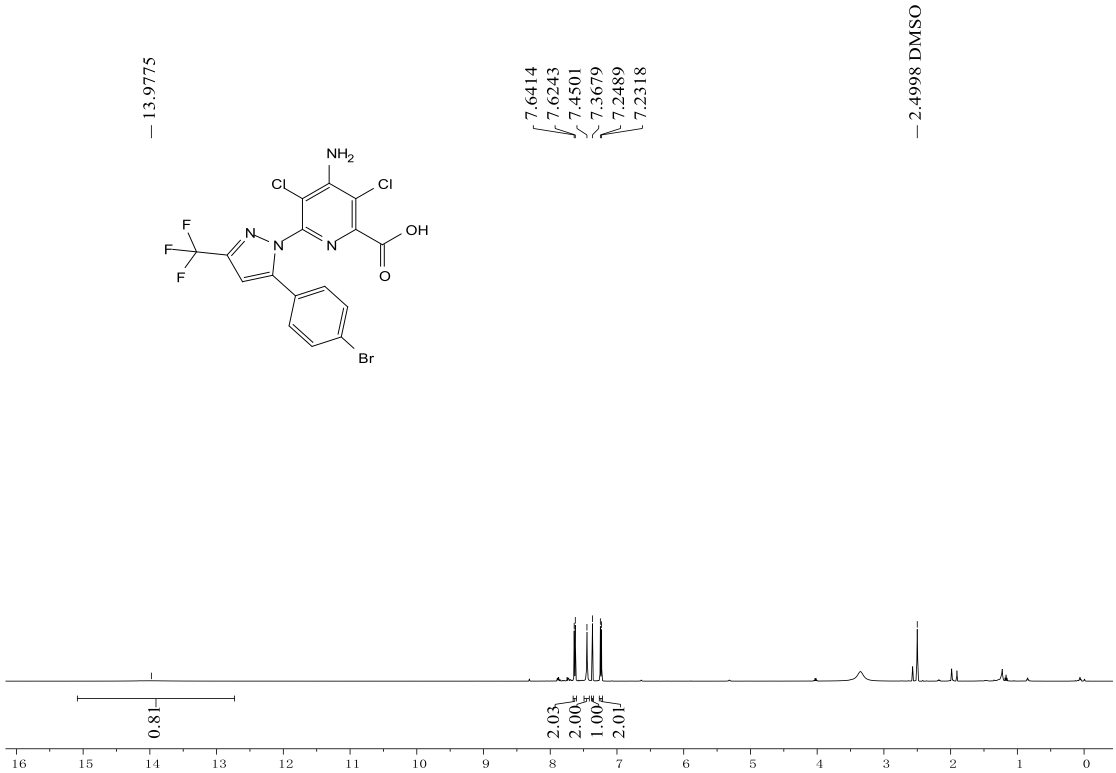

Figure S29. ^{1}H NMR spectrum of compound V-15.

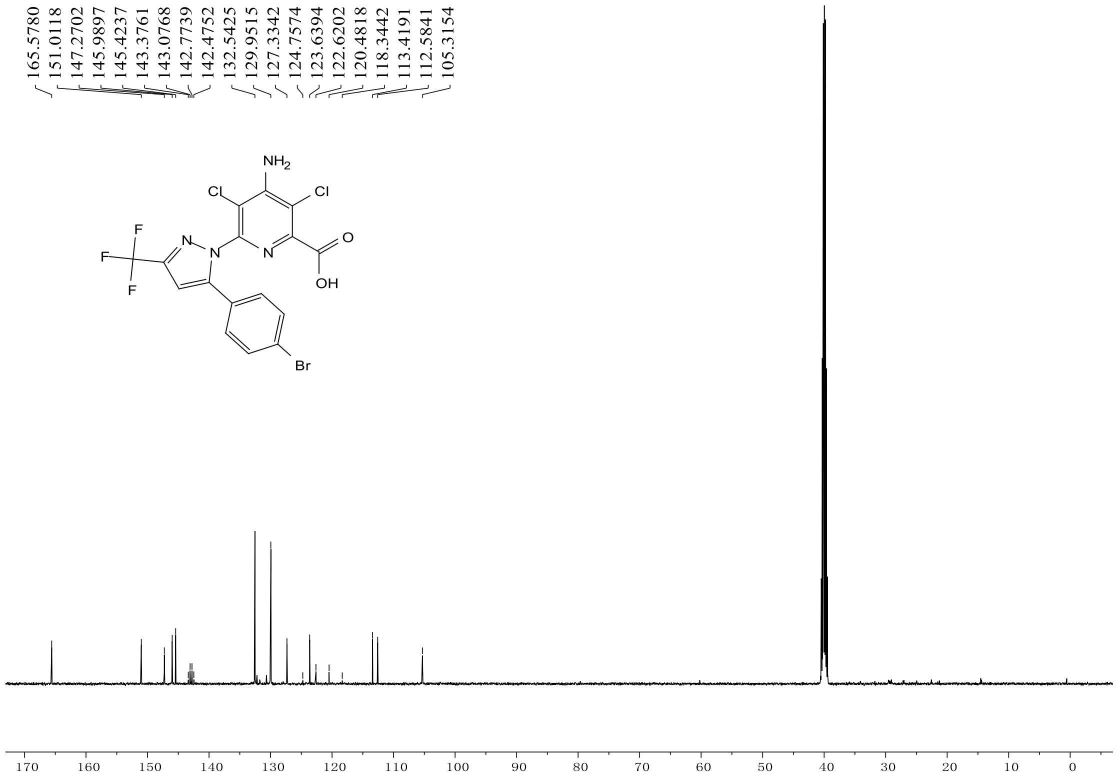

Figure S30. ^{13}C NMR spectrum of compound V-15.

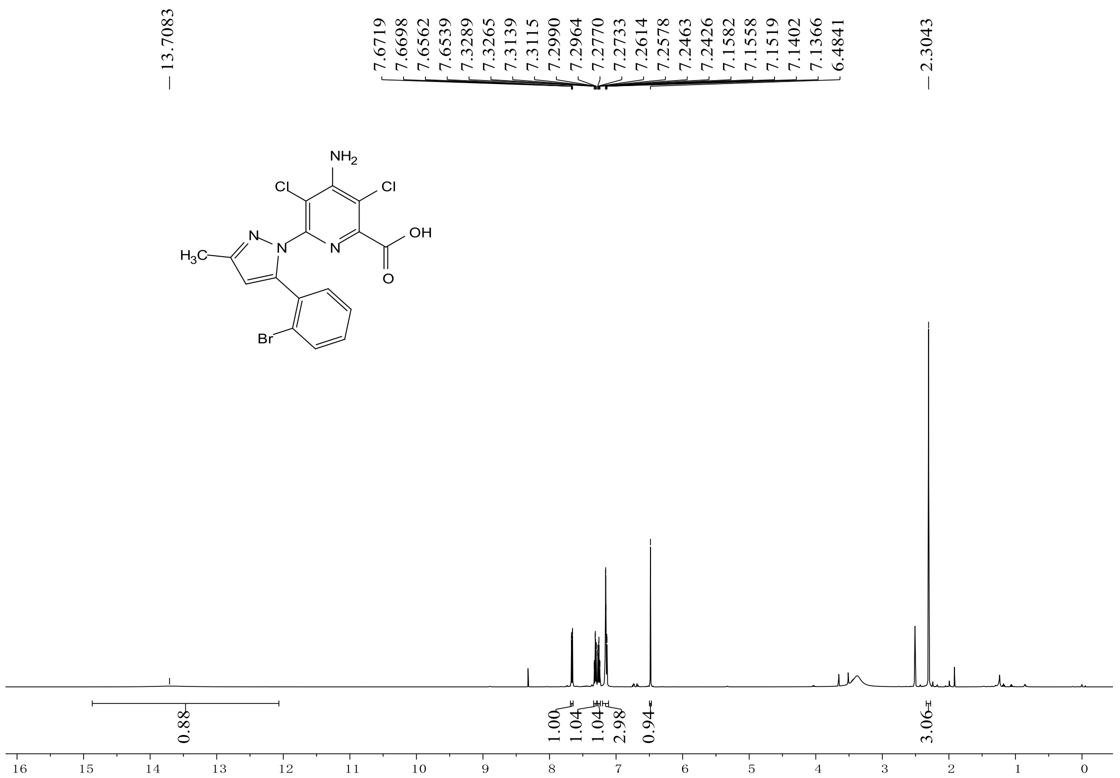

Figure S31. ^{1}H NMR spectrum of compound V-16.

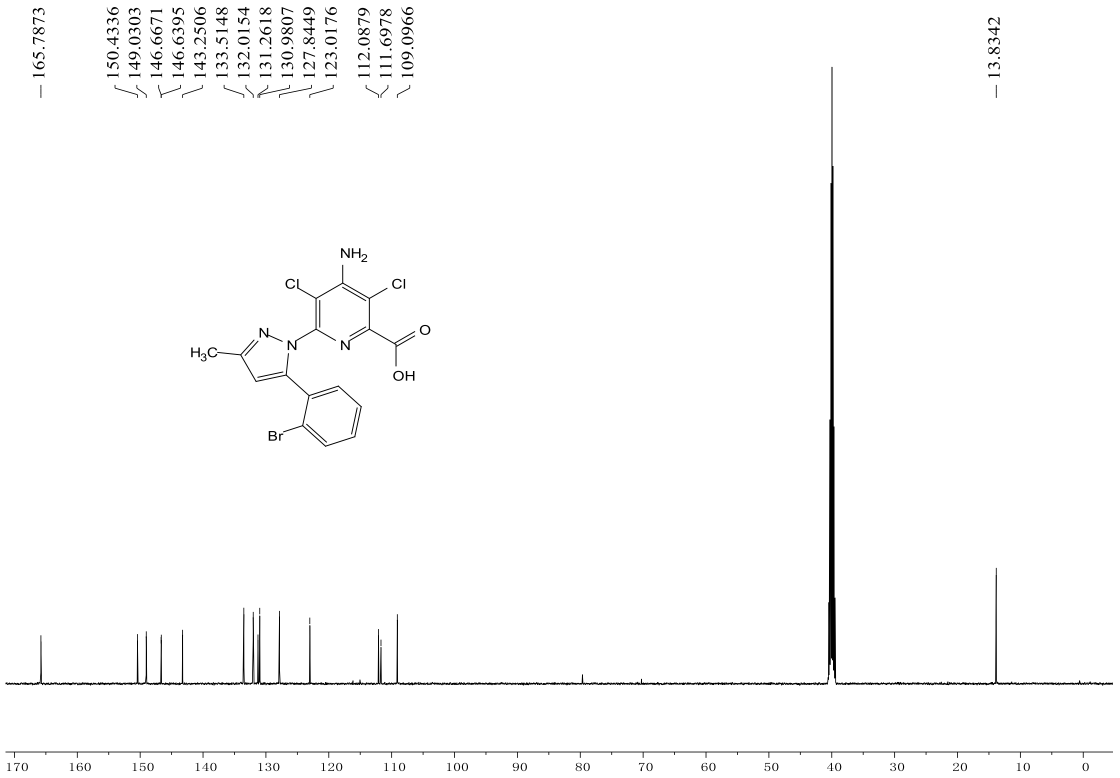

Figure S32. ^{13}C NMR spectrum of compound V-16.

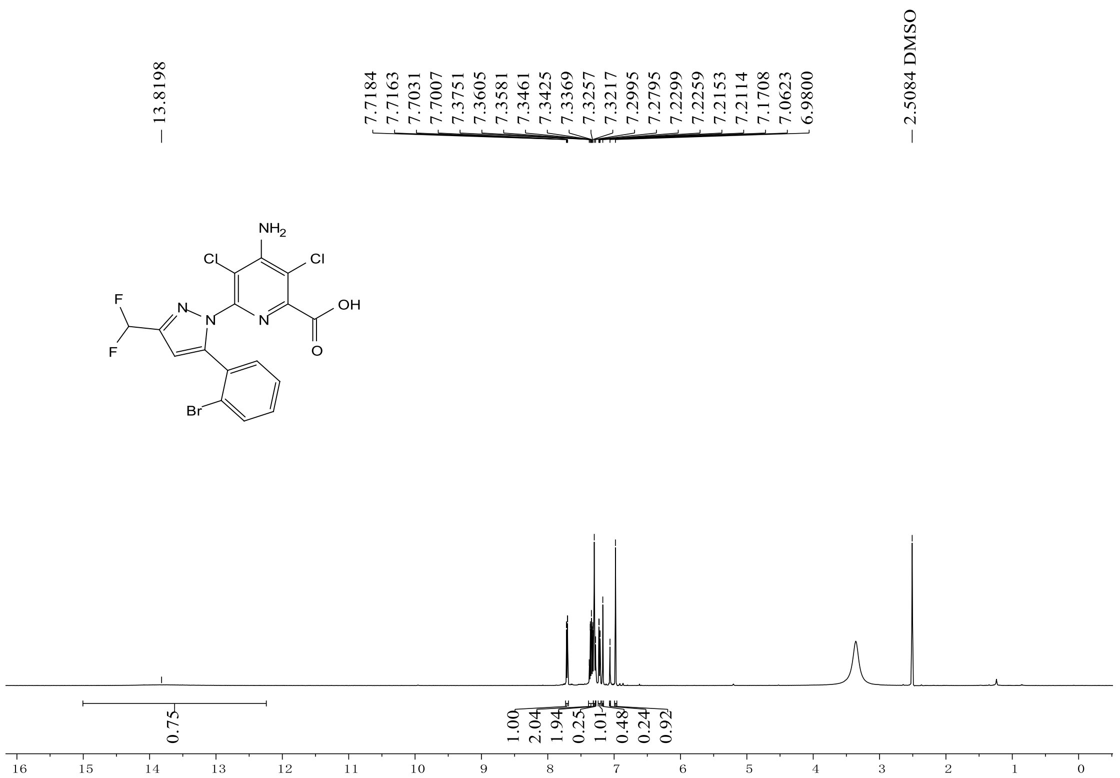

Figure S33. ^{1}H NMR spectrum of compound V-17.

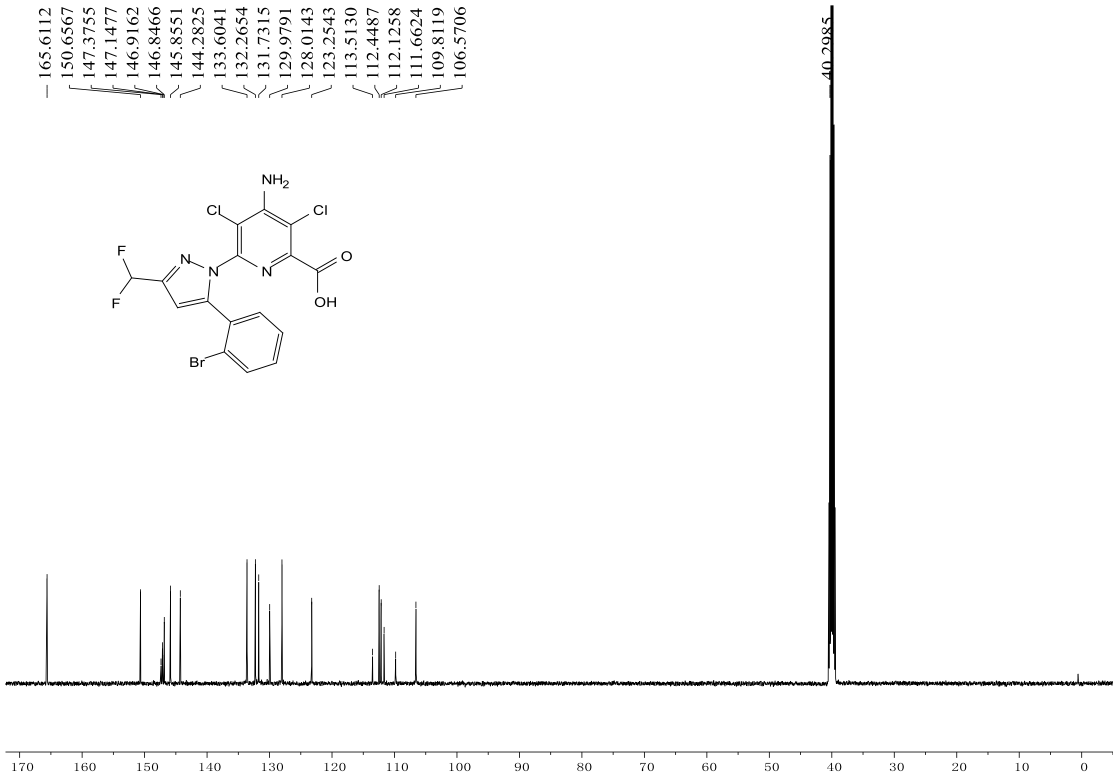

Figure S34. ^{13}C NMR spectrum of compound V-17.

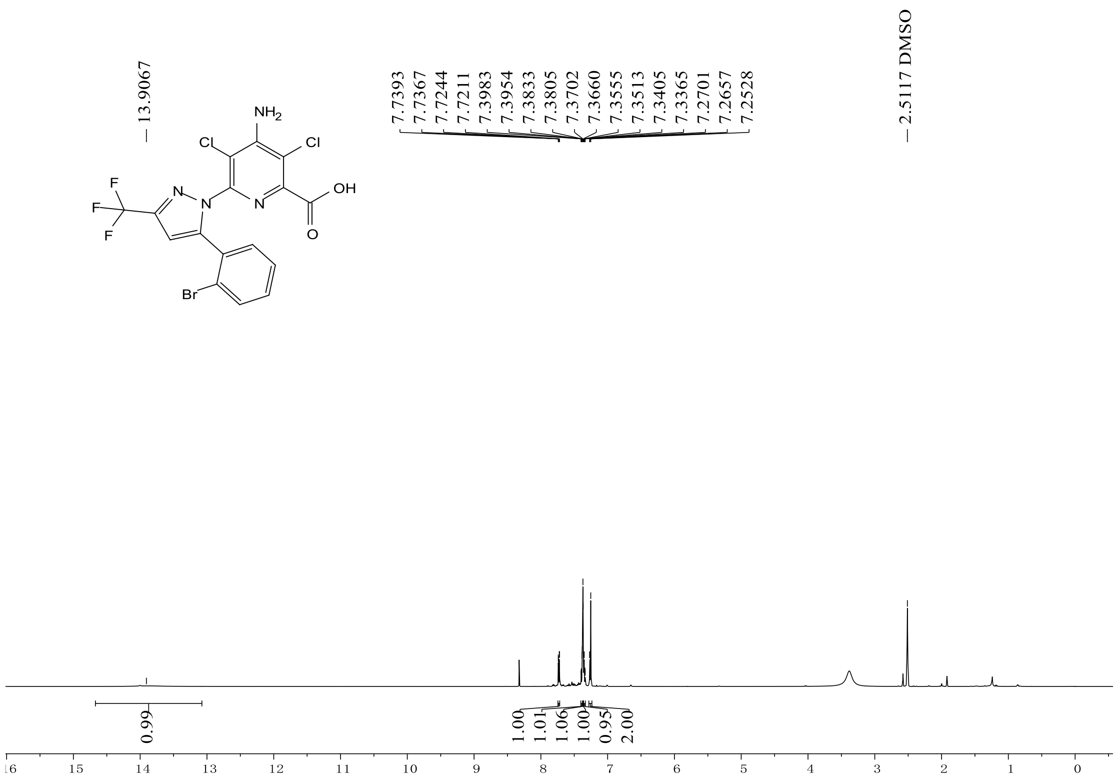

Figure S35. ^{1}H NMR spectrum of compound V-18.

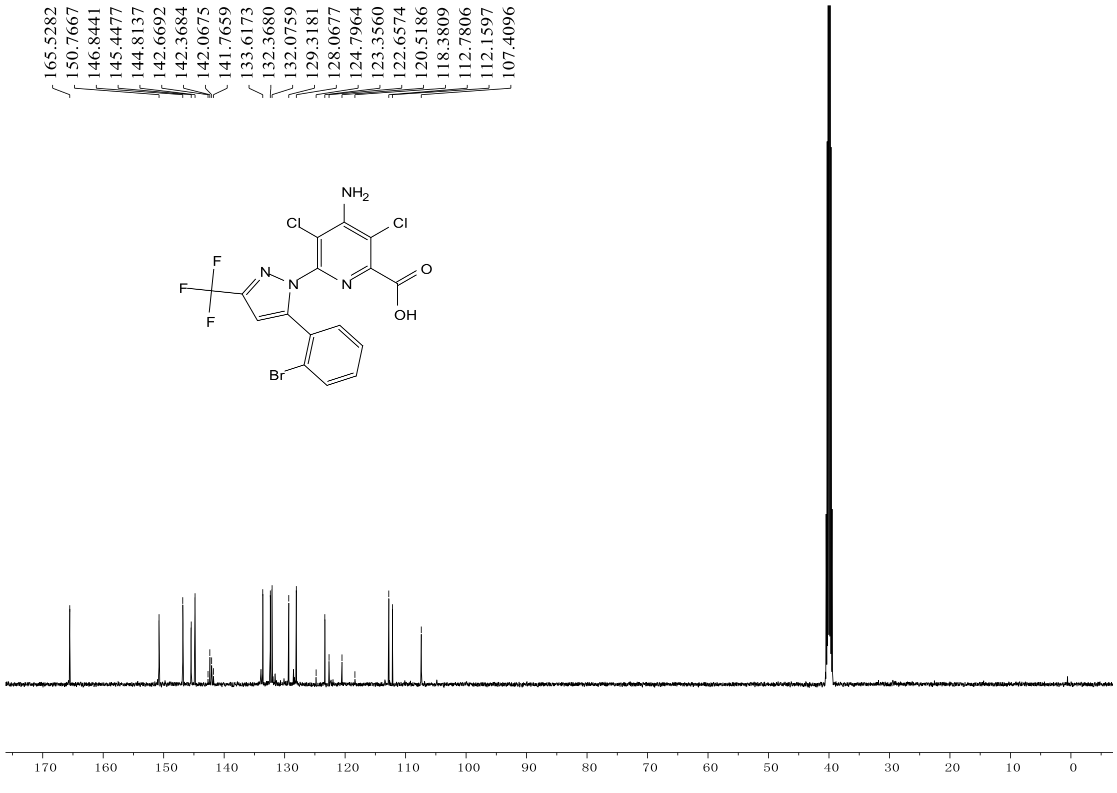

Figure S36. ^{13}C NMR spectrum of compound V-18.

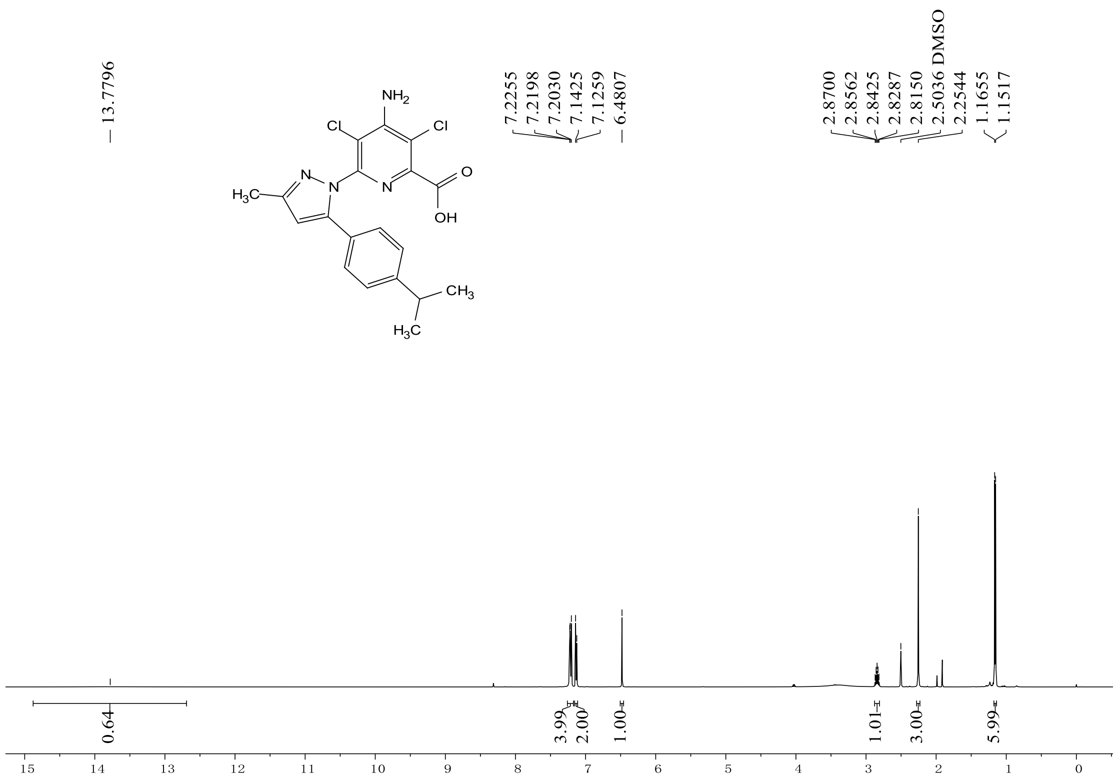

Figure S37. ^{1}H NMR spectrum of compound V-19.

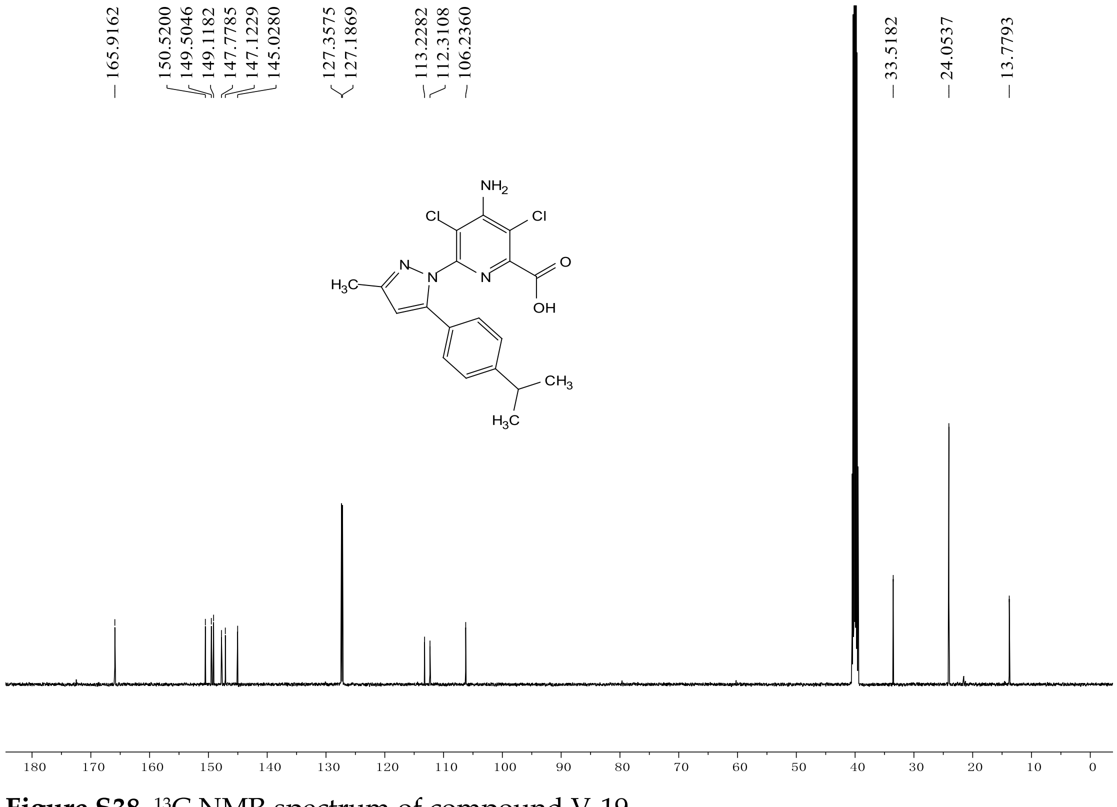

Figure S38. ^{13}C NMR spectrum of compound V-19.

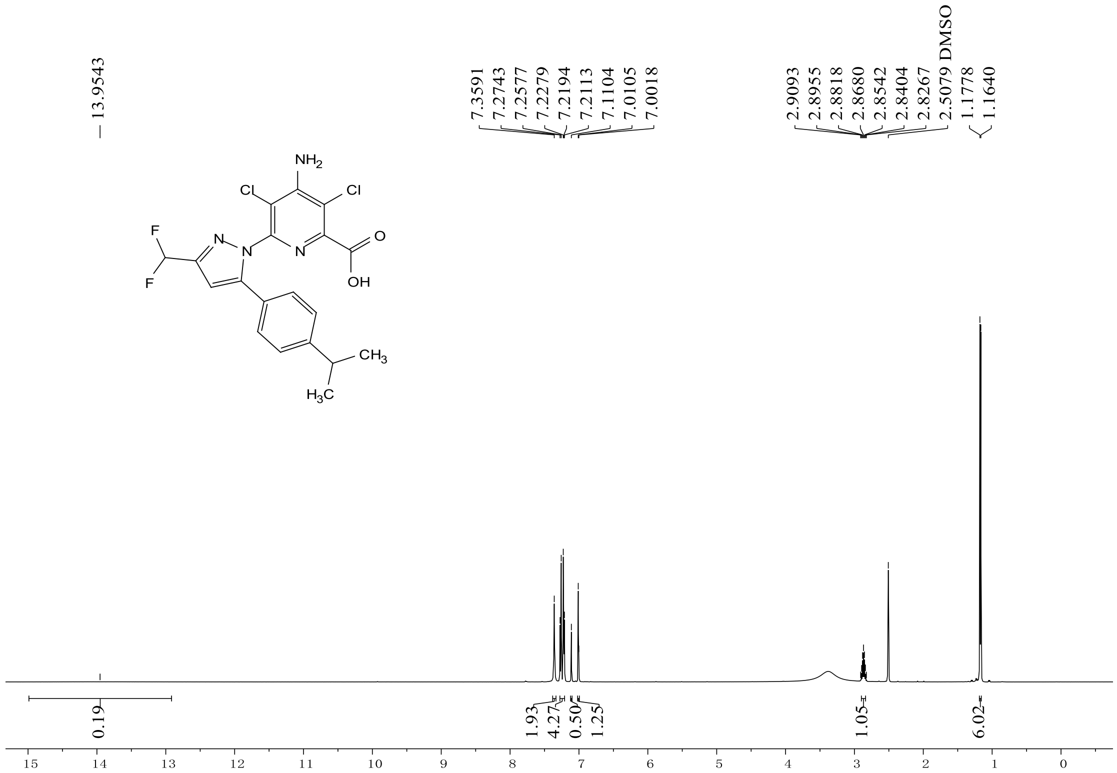

Figure S39. ^{1}H NMR spectrum of compound V-20.

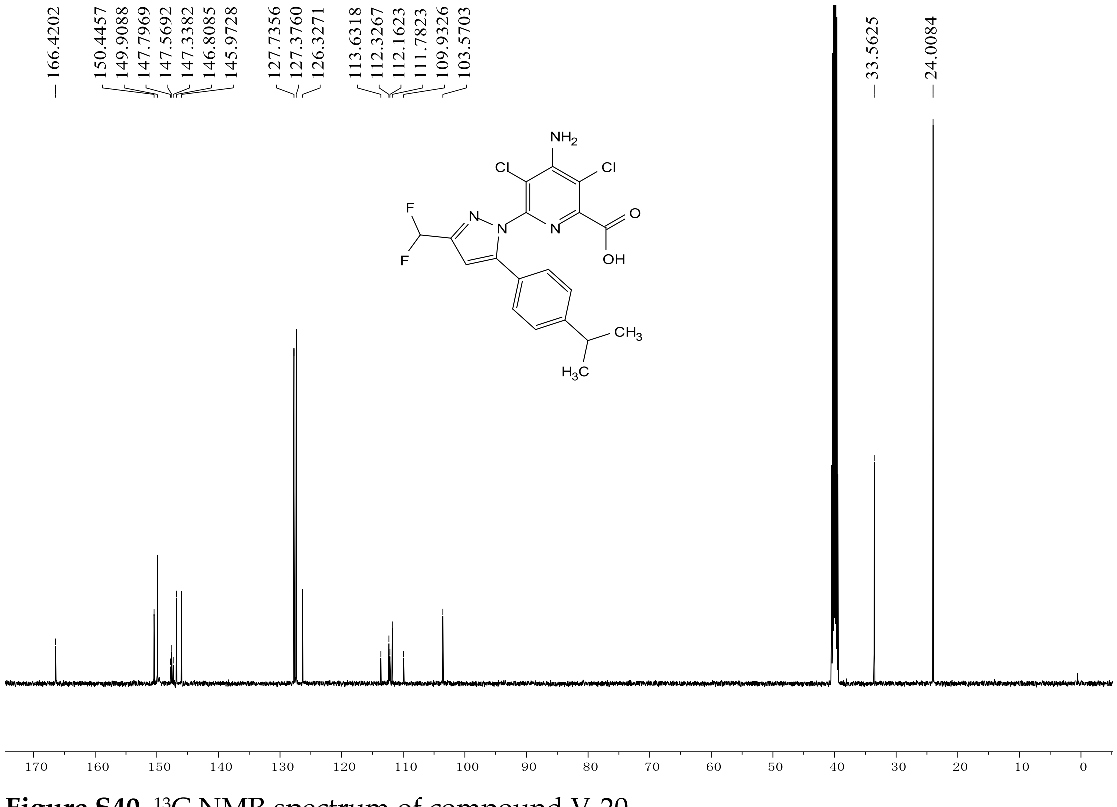

Figure S40. ^{13}C NMR spectrum of compound V-20.

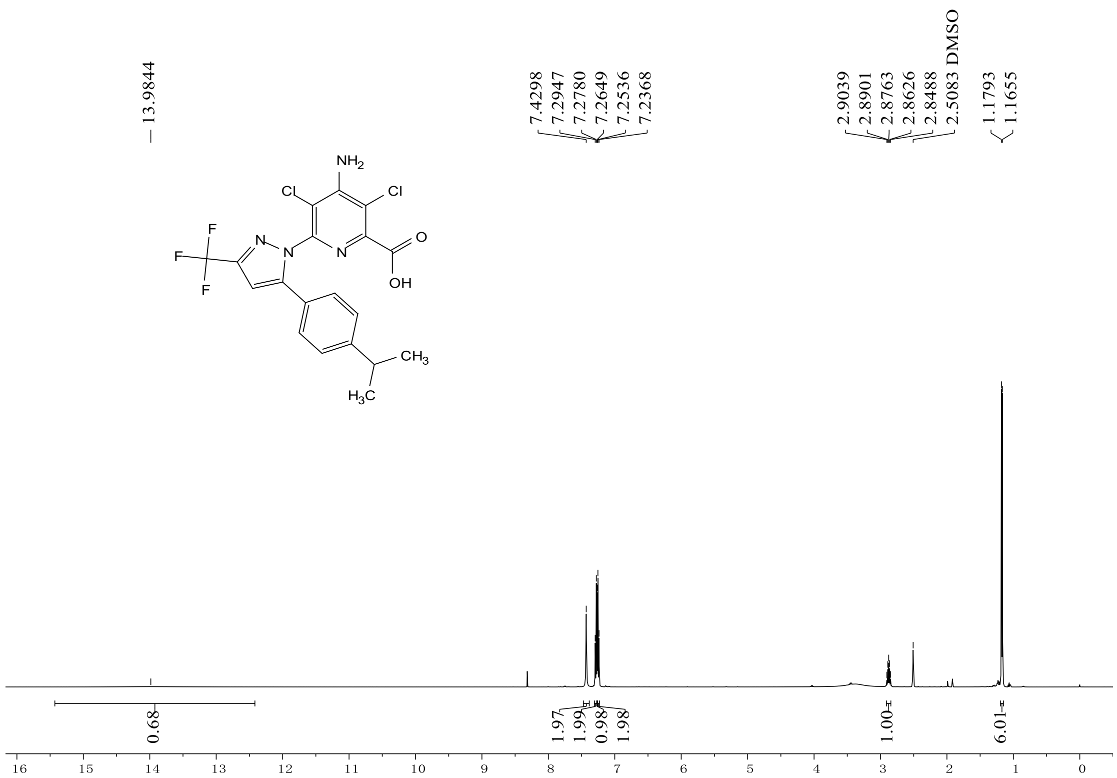

Figure S41. ^{1}H NMR spectrum of compound V-21.

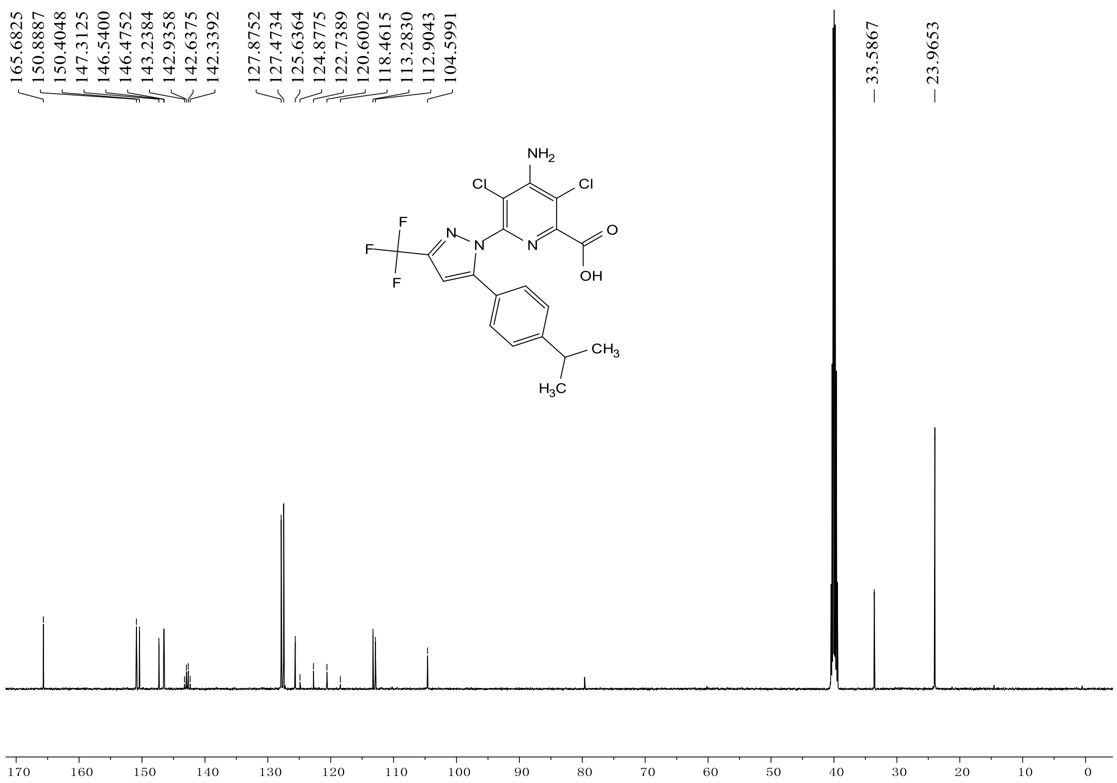

Figure S42. ^{13}C NMR spectrum of compound V-21.

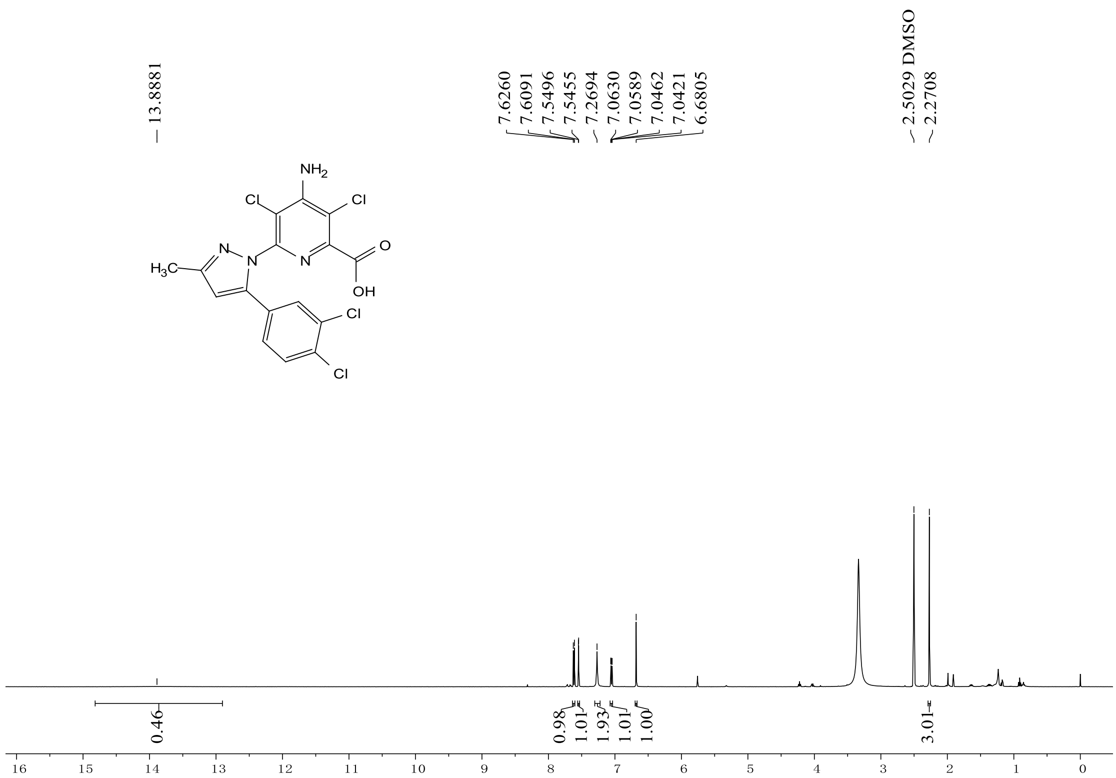

Figure S43. ^{1}H NMR spectrum of compound V-22.

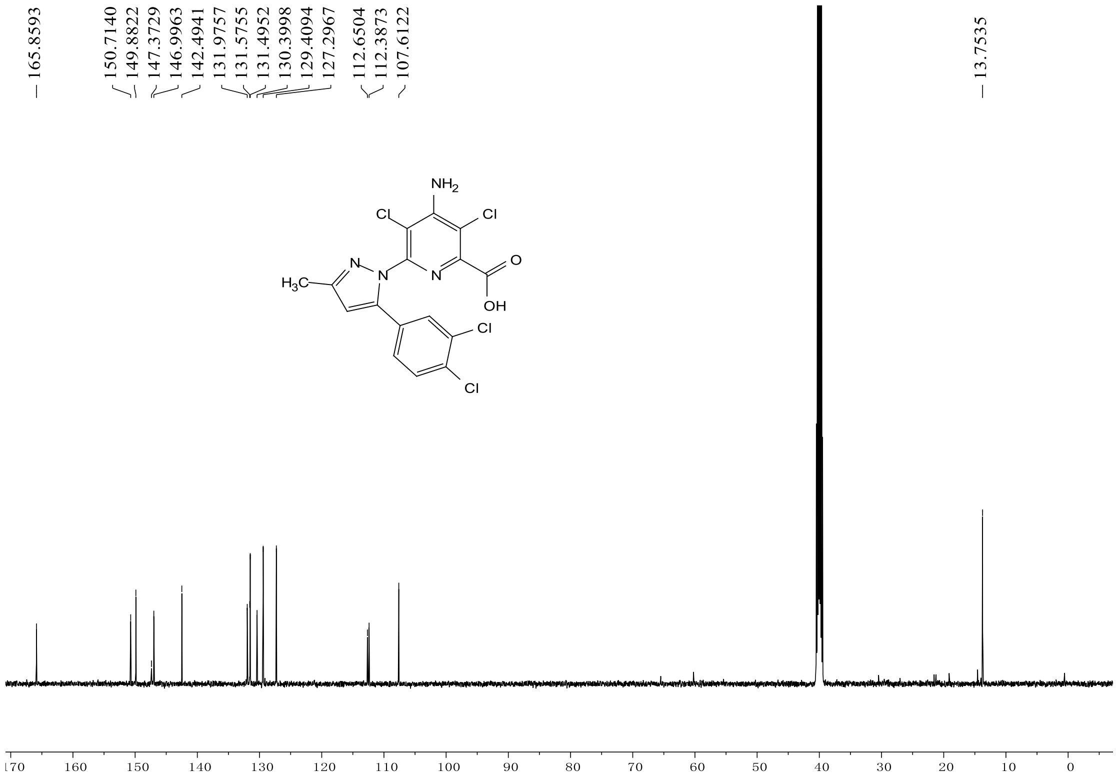

Figure S44. ^{13}C NMR spectrum of compound V-22.

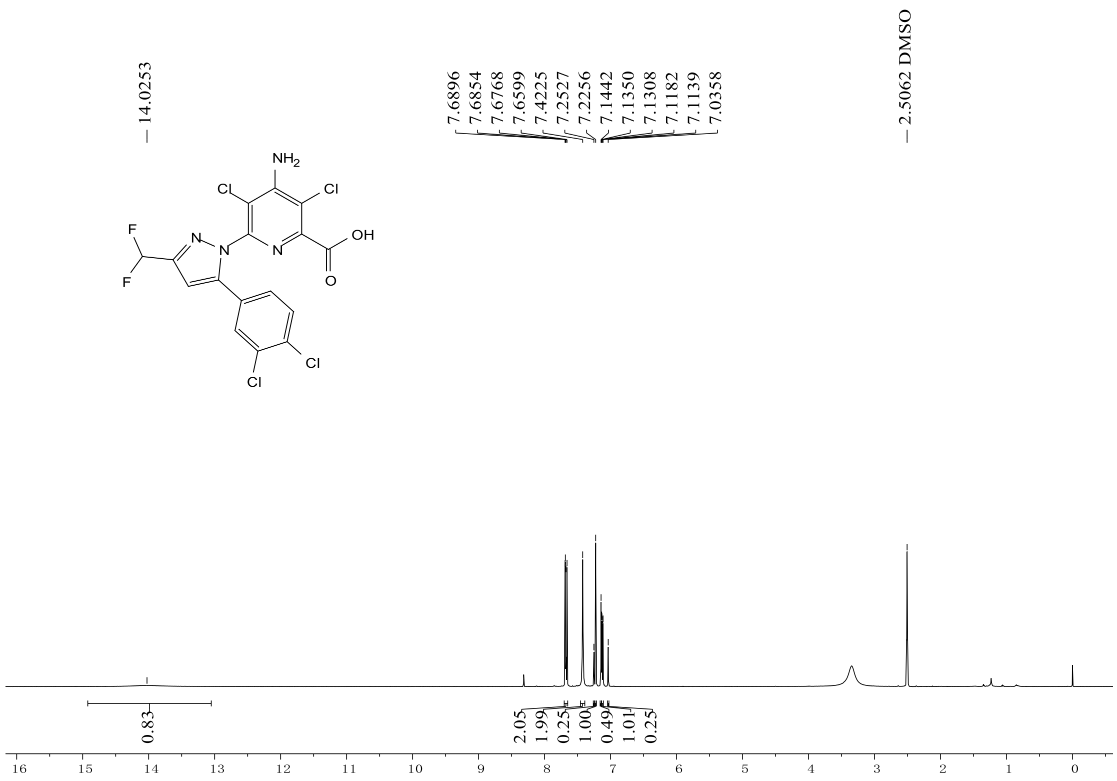

Figure S45. ^{1}H NMR spectrum of compound V-23.

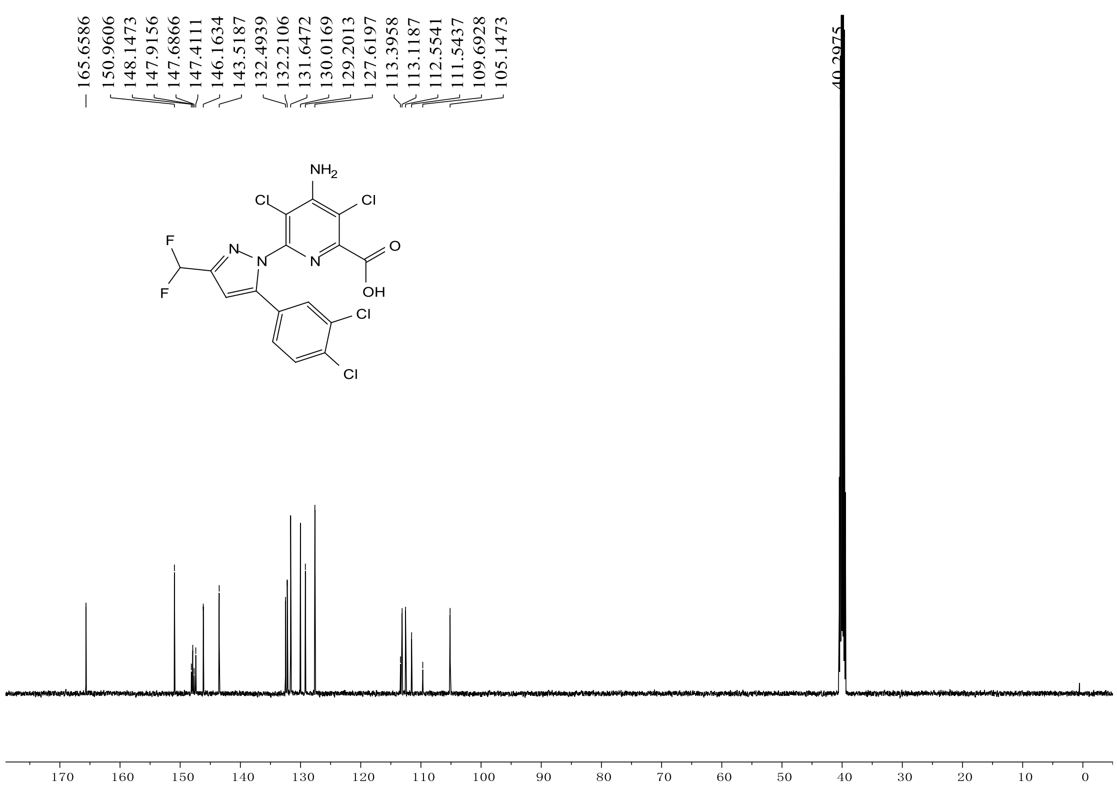

Figure S46. ^{13}C NMR spectrum of compound V-23.

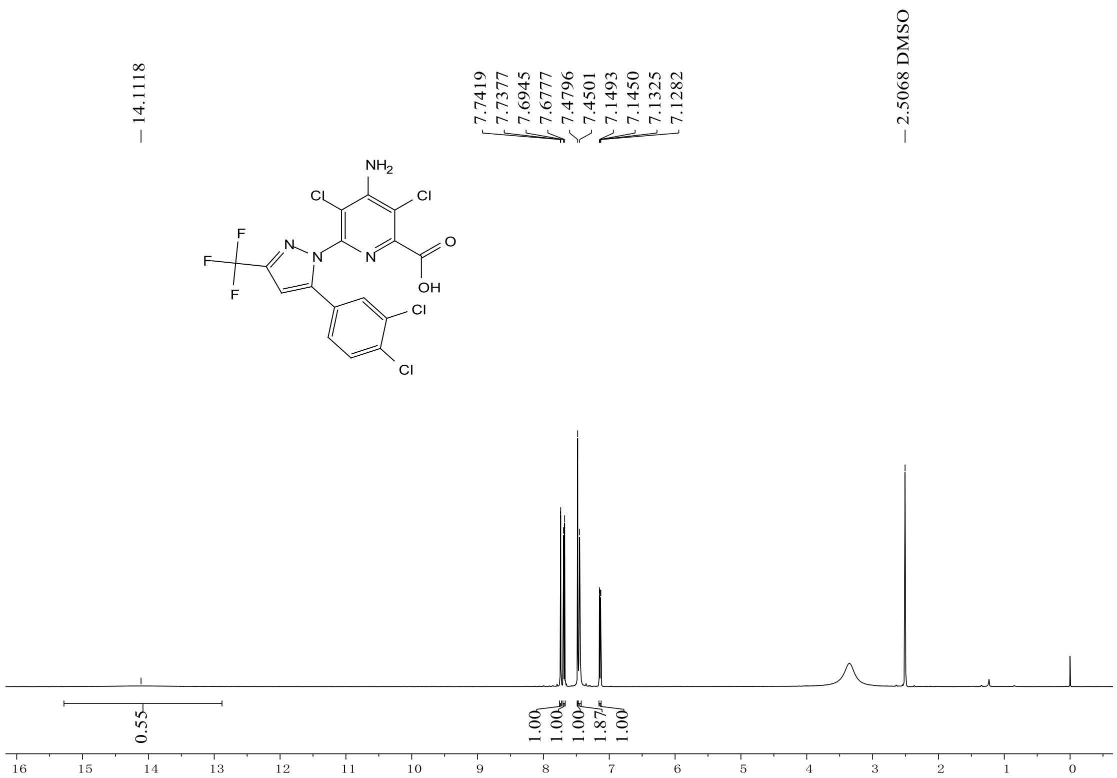

Figure S47. ^{1}H NMR spectrum of compound V-24.

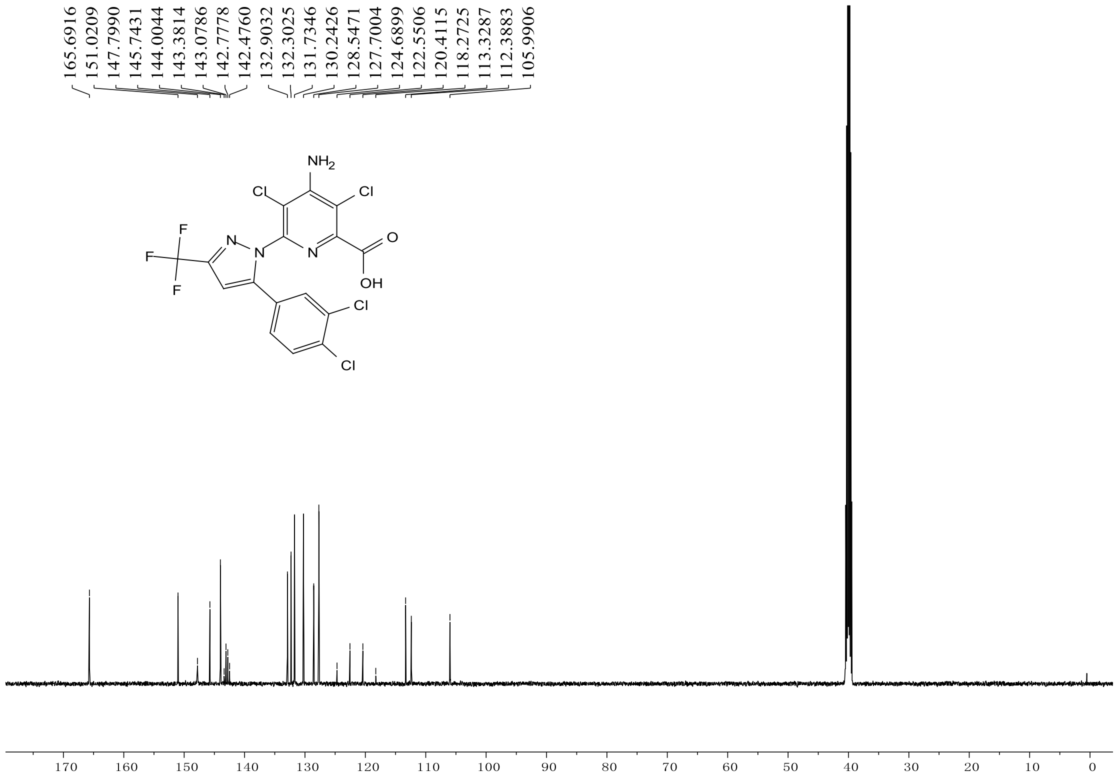

Figure S48. ^{13}C NMR spectrum of compound V-24.

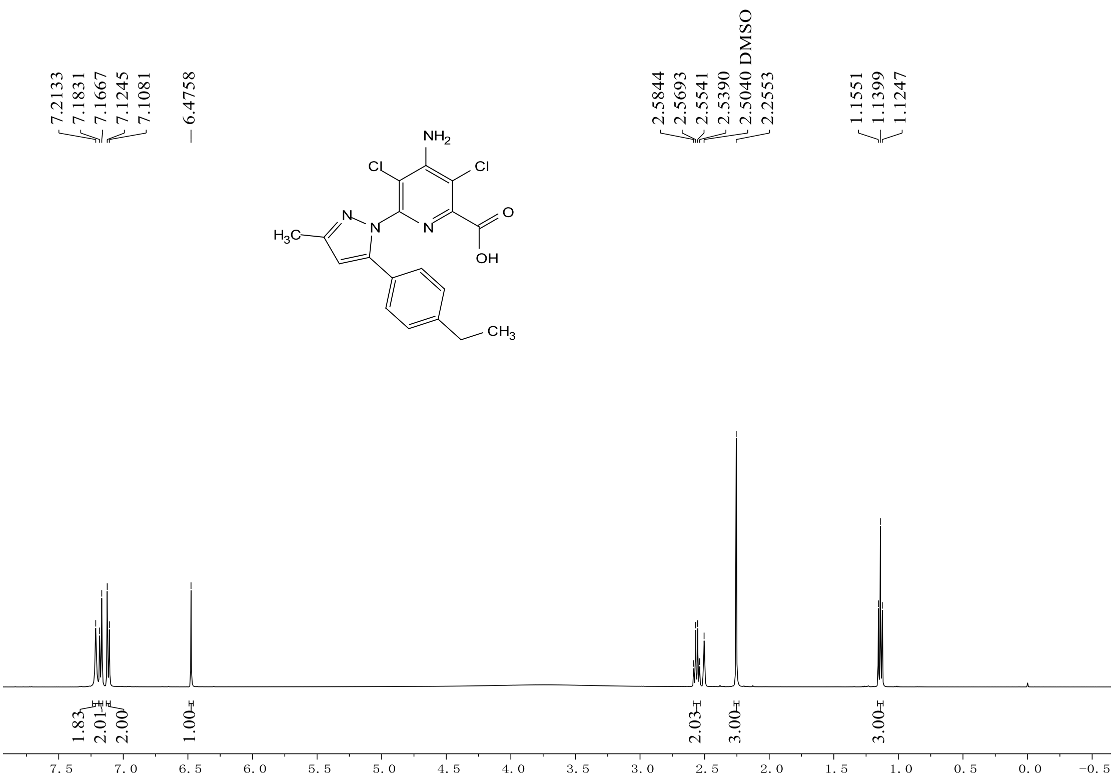

Figure S49. ^{1}H NMR spectrum of compound V-25.

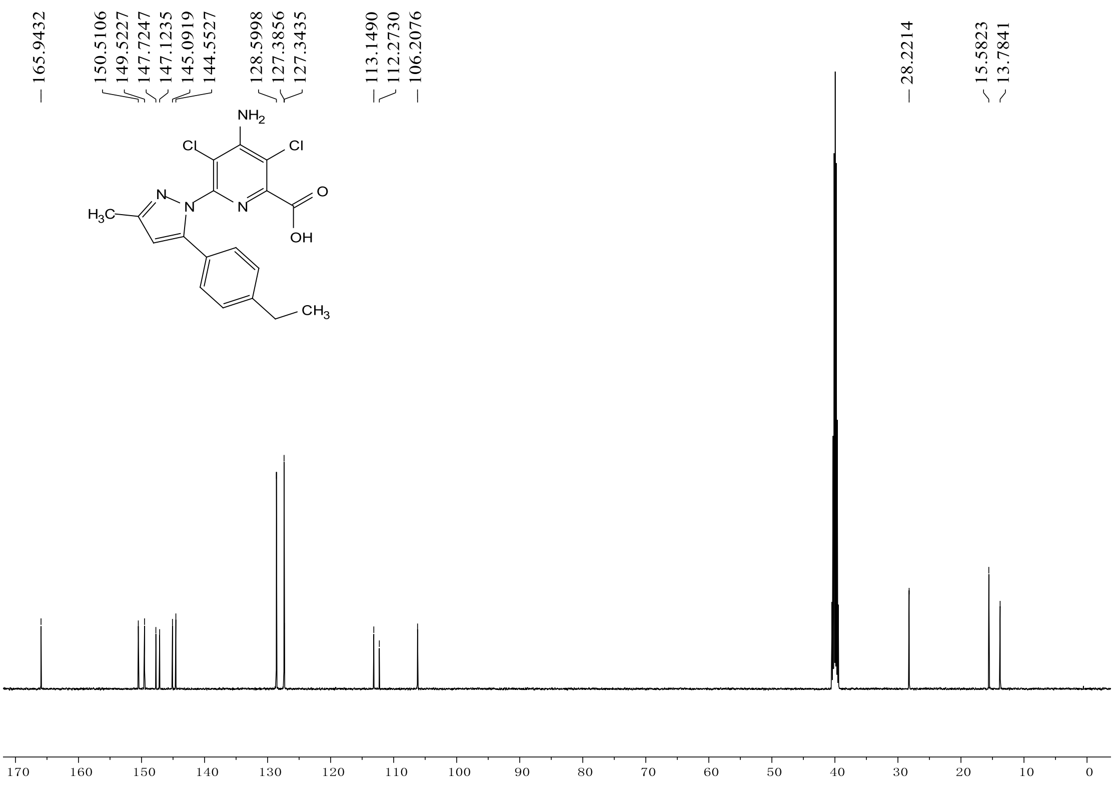

Figure S50. ^{13}C NMR spectrum of compound V-25.

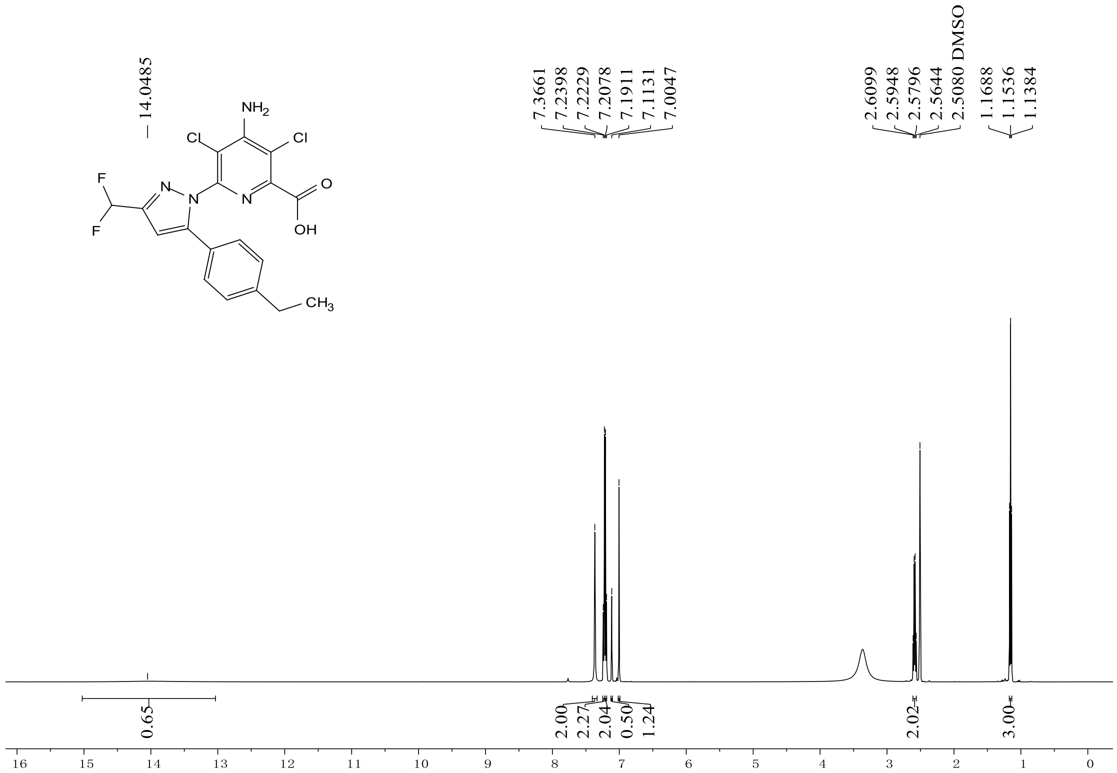

Figure S51. ^{1}H NMR spectrum of compound V-26.

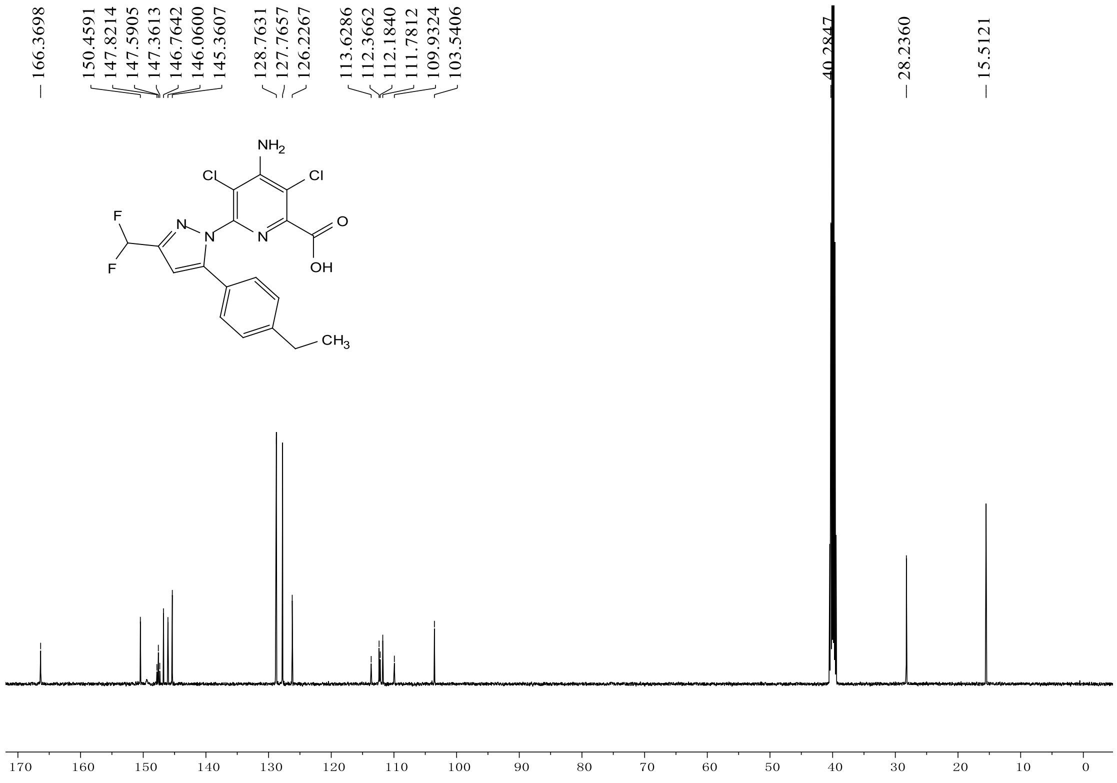

Figure S52. ^{13}C NMR spectrum of compound V-26.

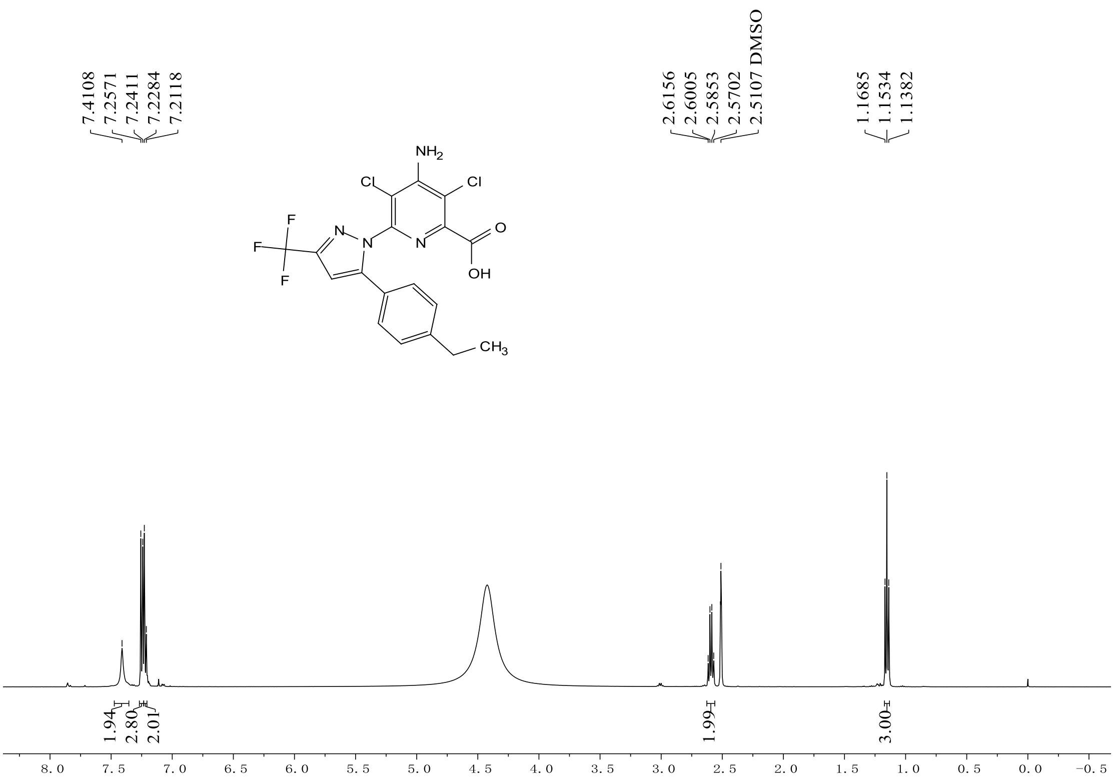

Figure S53. ^1H NMR spectrum of compound V-27.

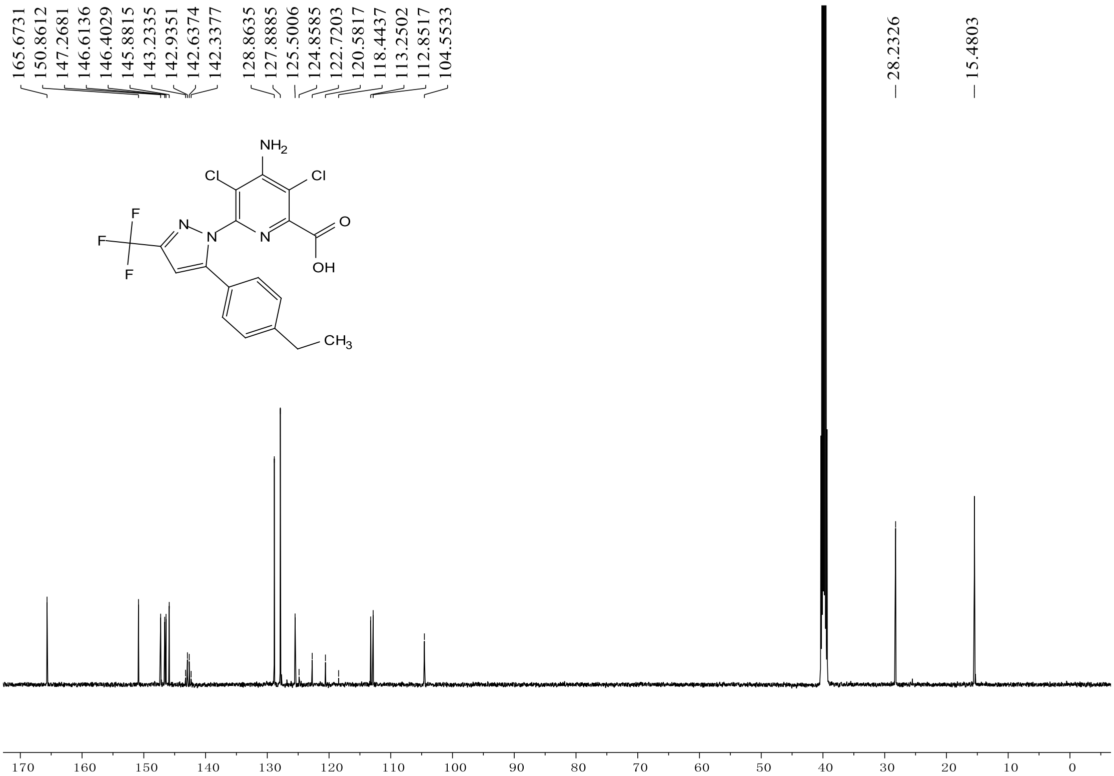

Figure S54. ^{13}C NMR spectrum of compound V-27.

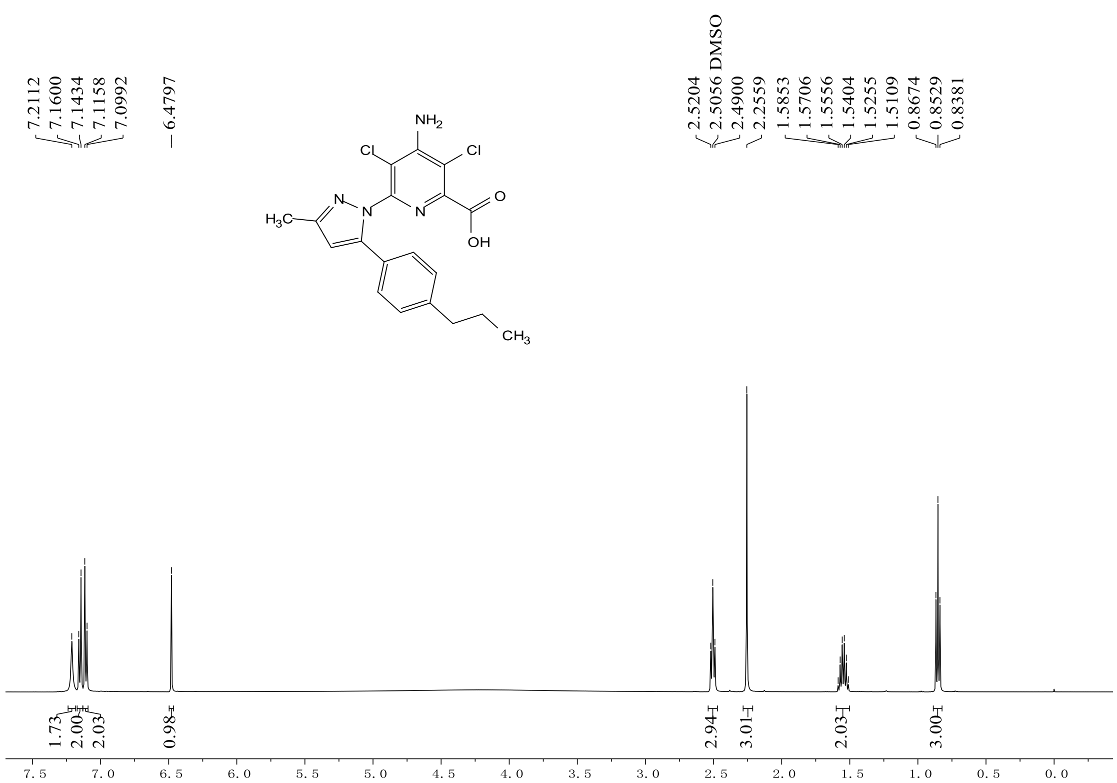

Figure S55. ^{1}H NMR spectrum of compound V-28.

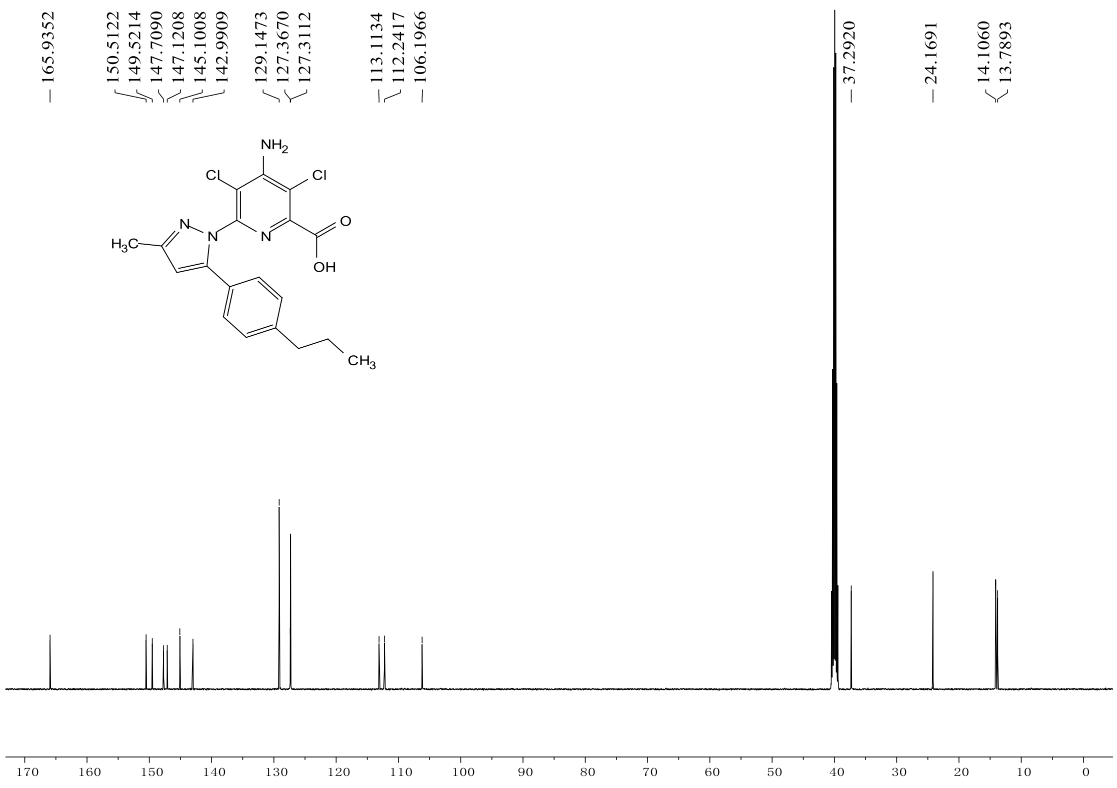

Figure S56. ^{13}C NMR spectrum of compound V-28.

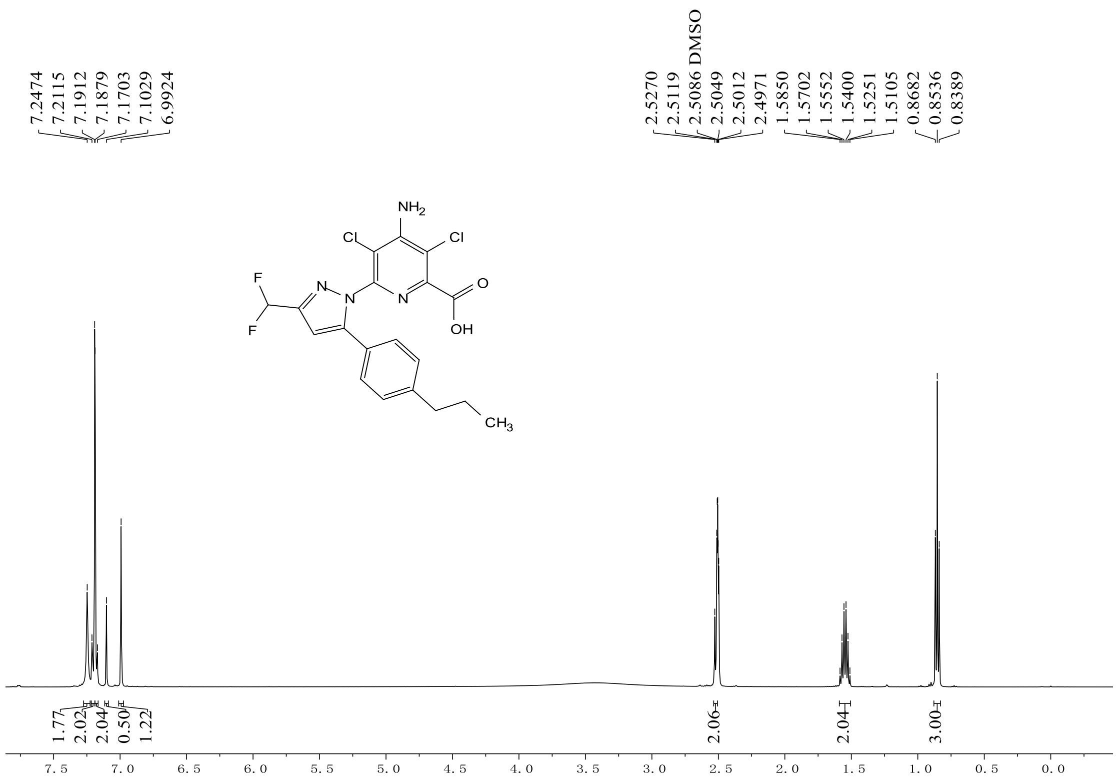

Figure S57. ^{1}H NMR spectrum of compound V-29.

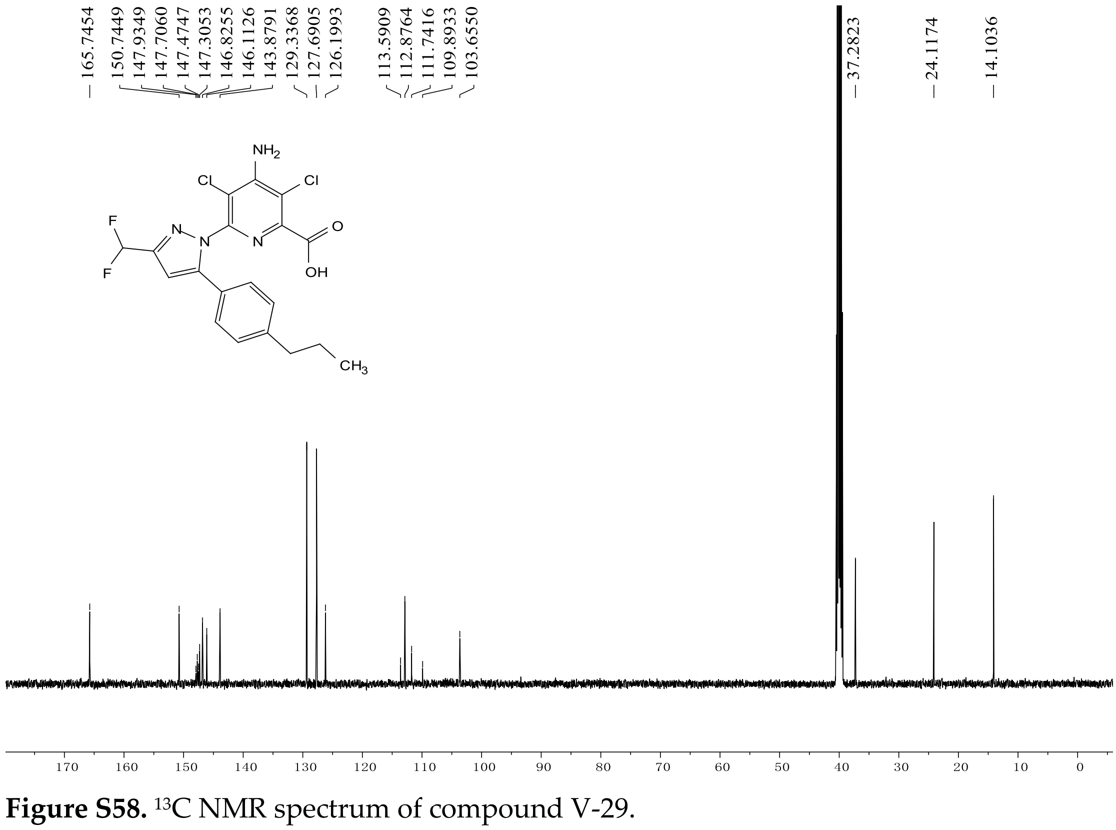

Figure S58. ^{13}C NMR spectrum of compound V-29.

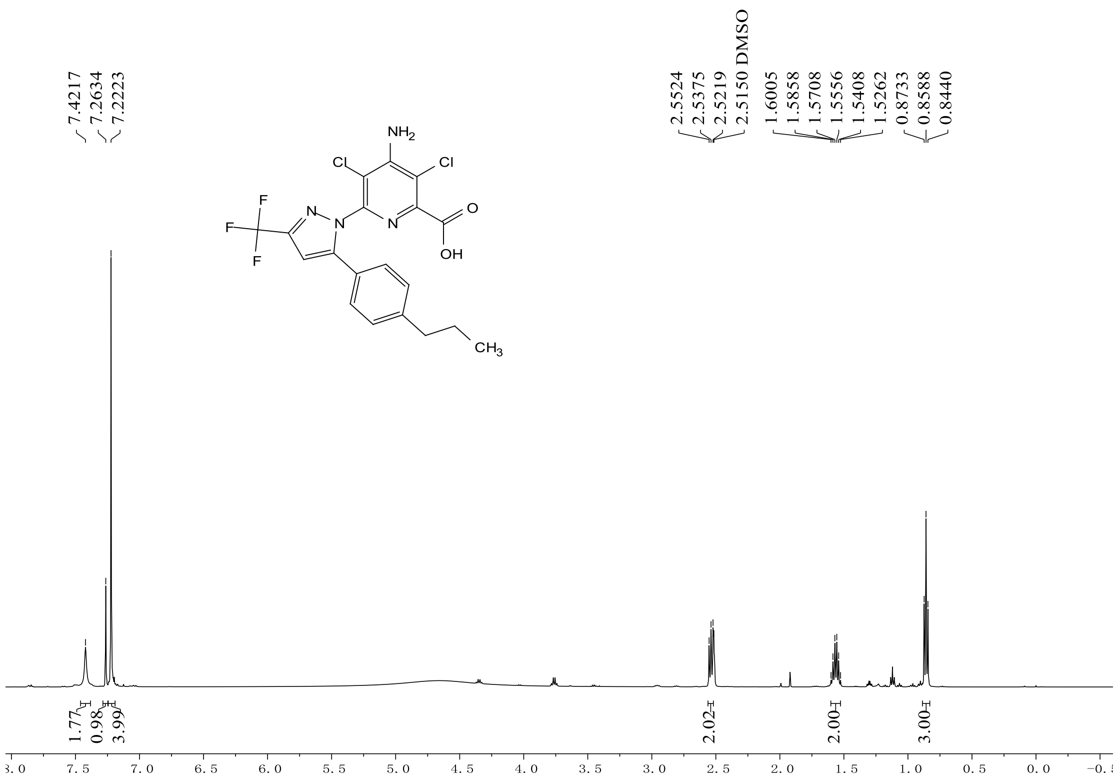

Figure S59. ^{1}H NMR spectrum of compound V-30.

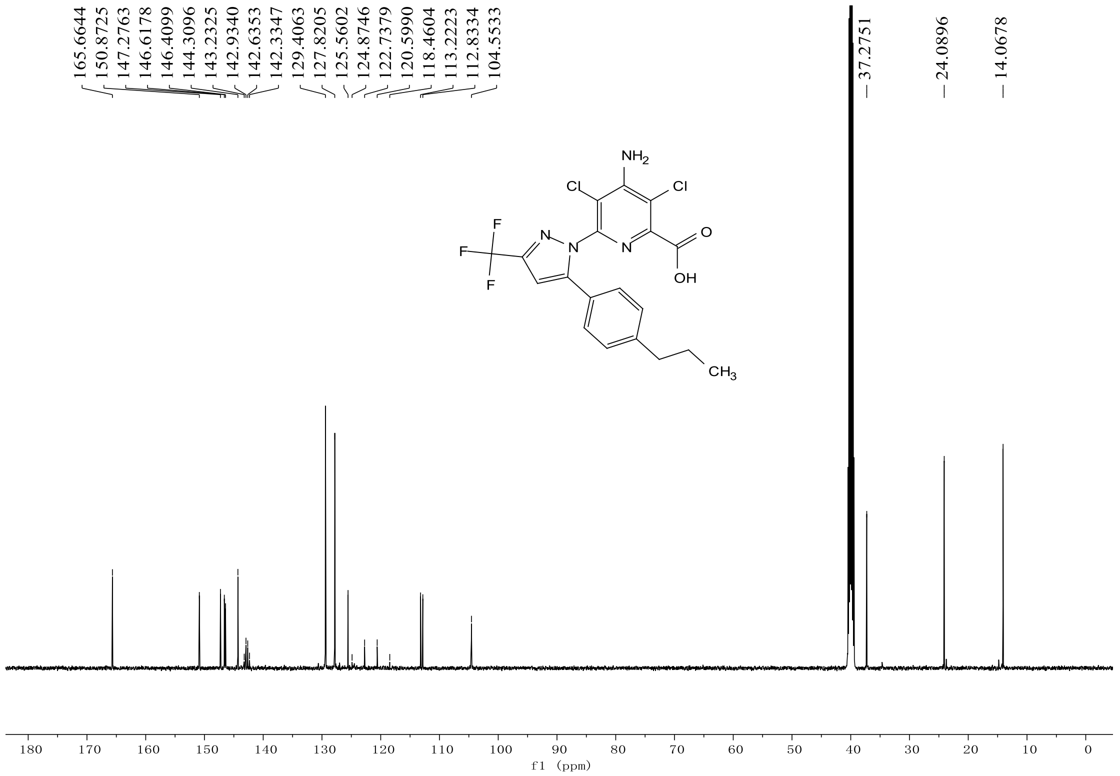

Figure S60. ^{13}C NMR spectrum of compound V-30.

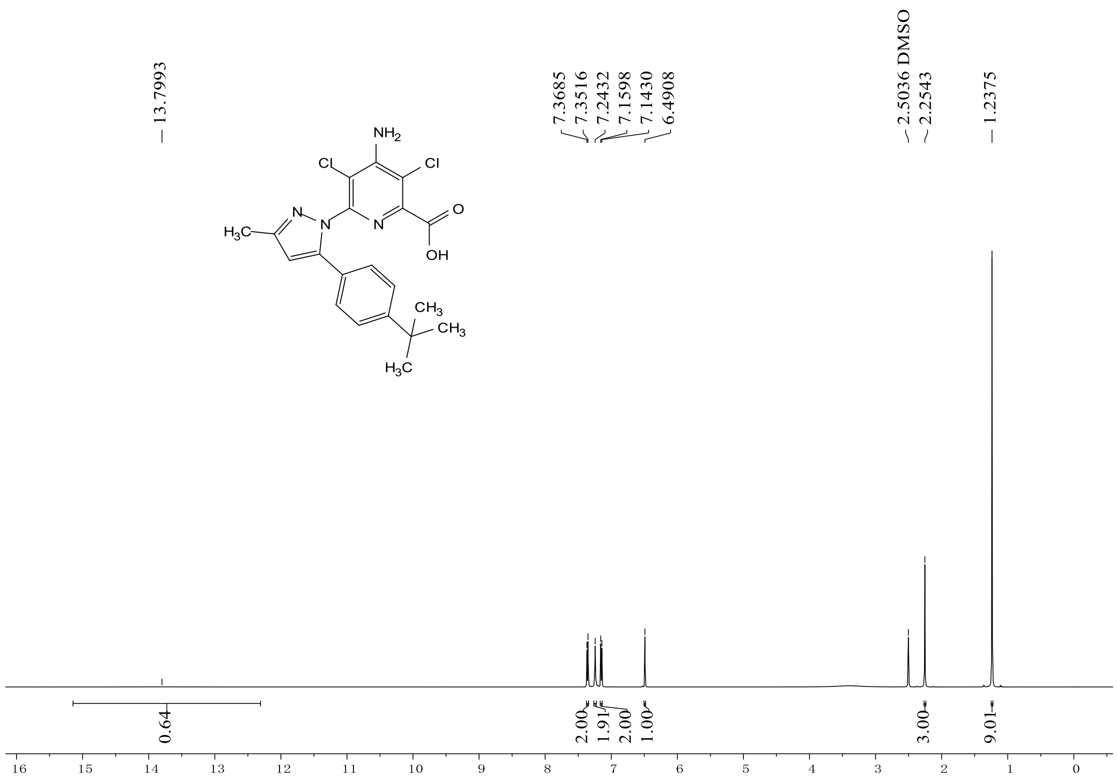

Figure S61. ^{1}H NMR spectrum of compound V-31.

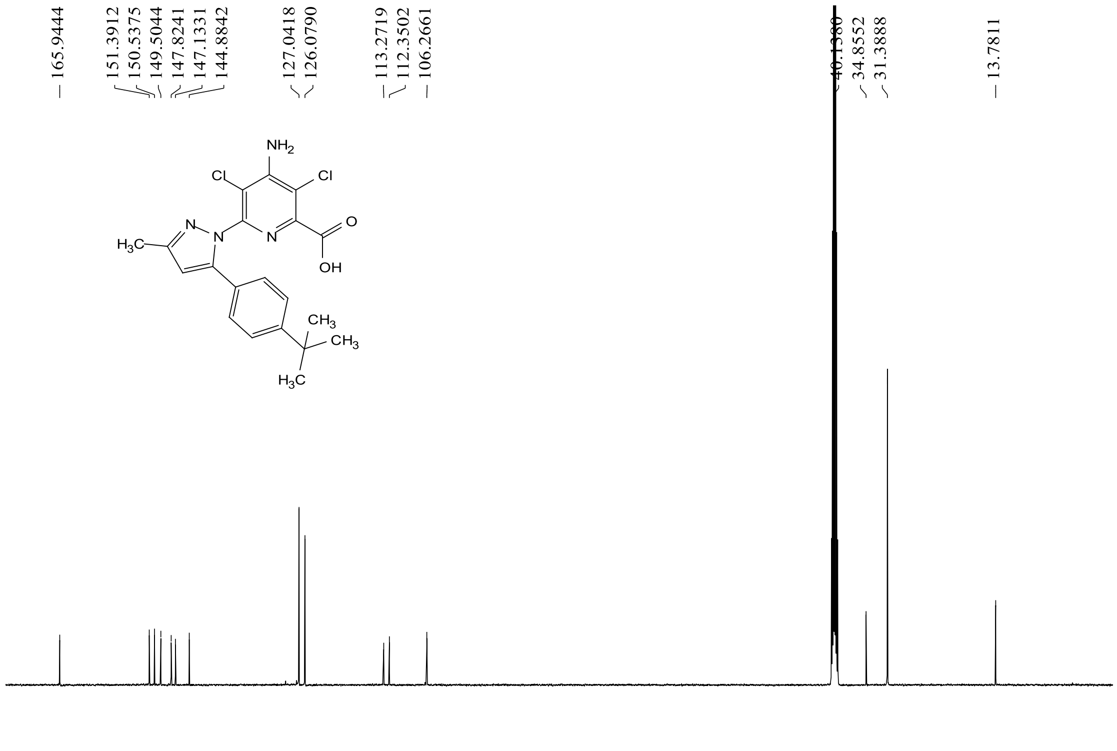

Figure S62. ^{13}C NMR spectrum of compound V-31.

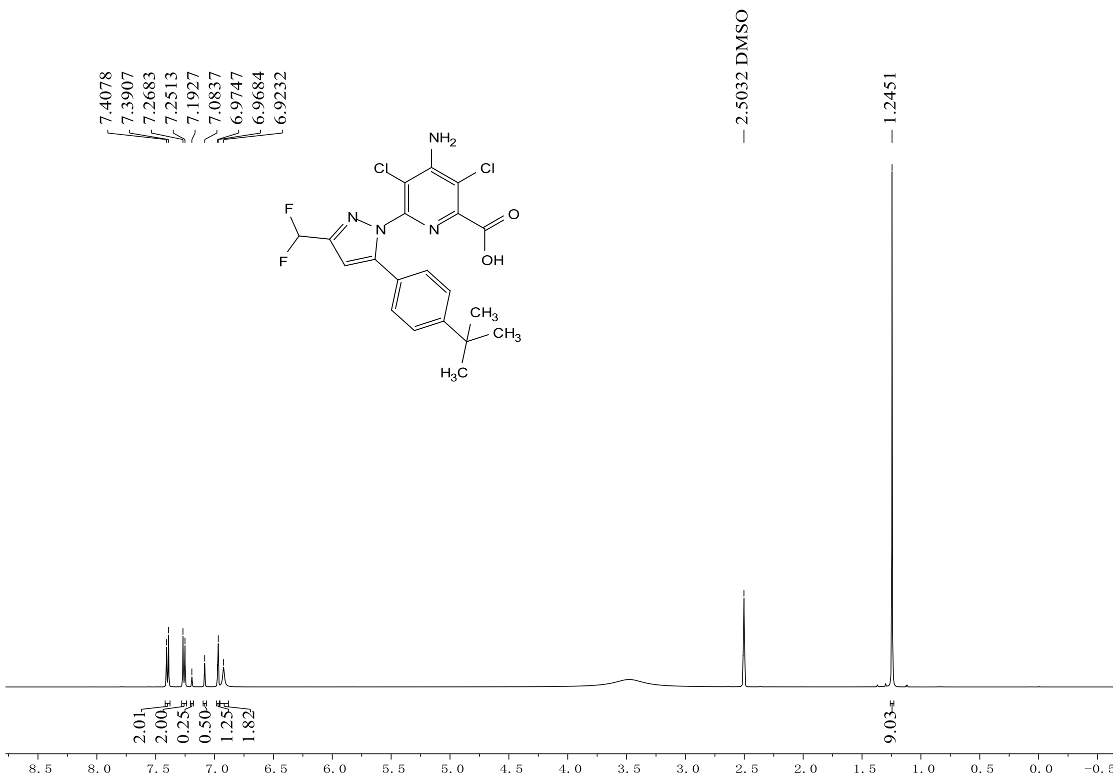

Figure S63. ^{1}H NMR spectrum of compound V-32.

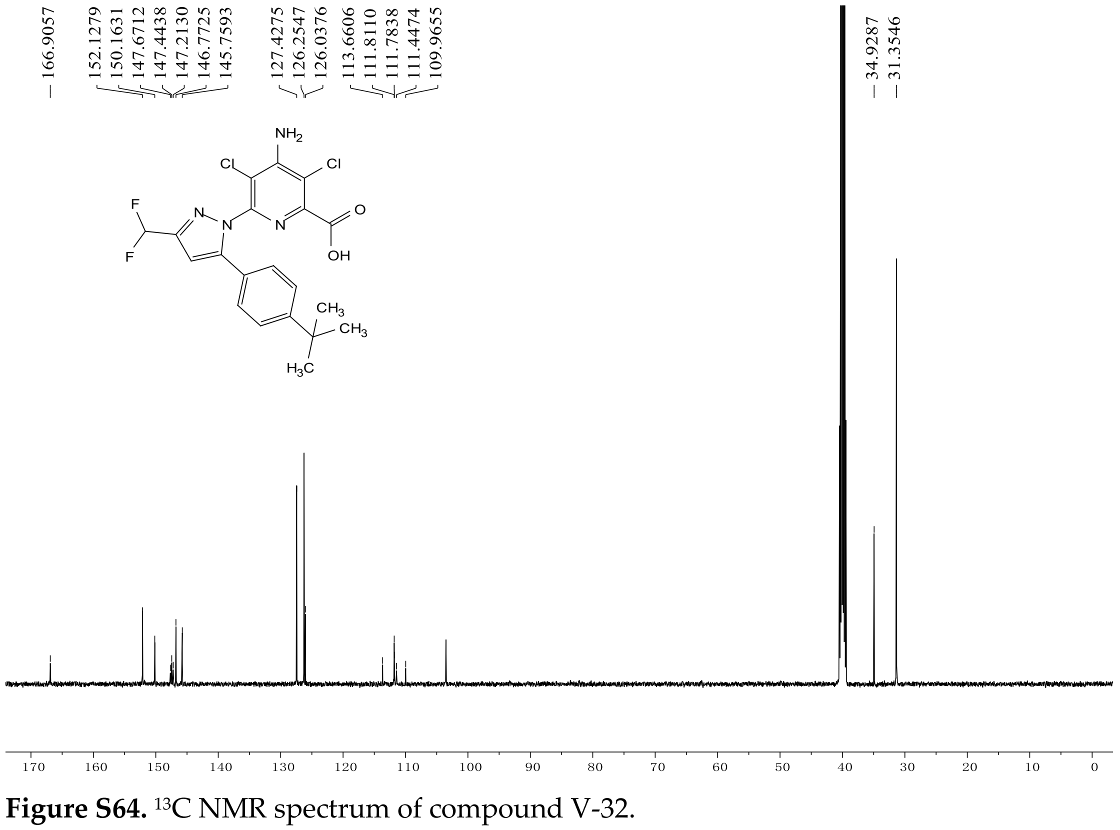

Figure S64. ^{13}C NMR spectrum of compound V-32.

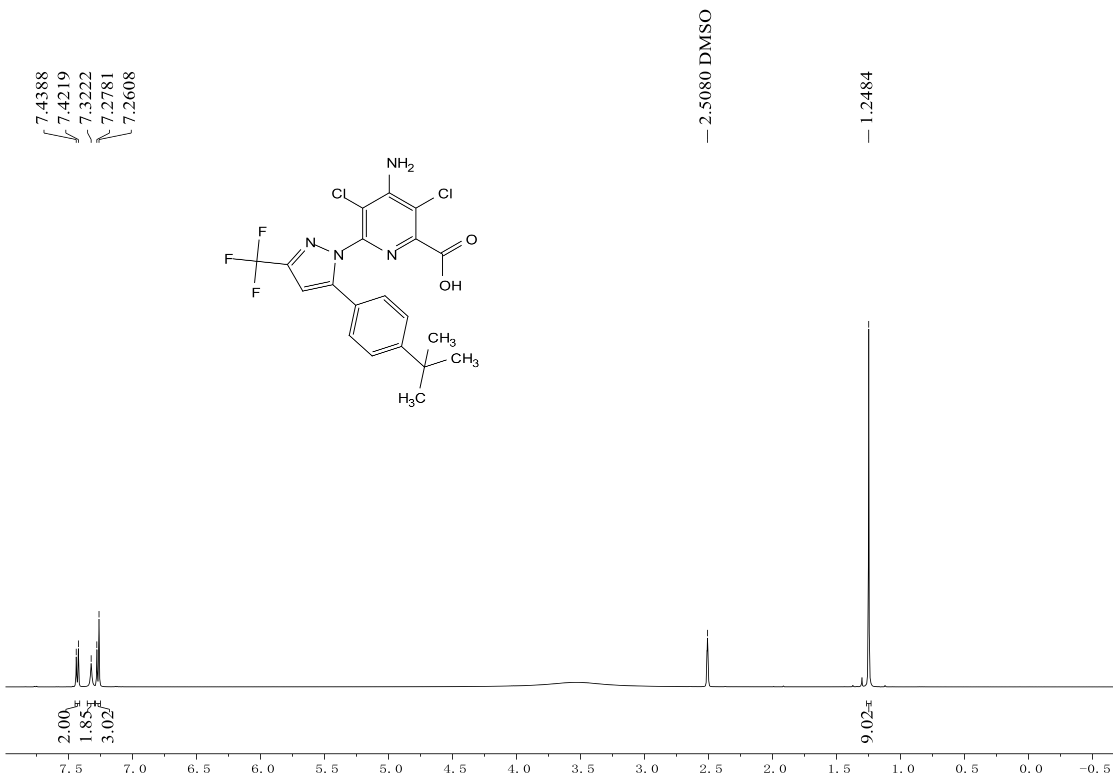

Figure S65. ^{1}H NMR spectrum of compound V-33.

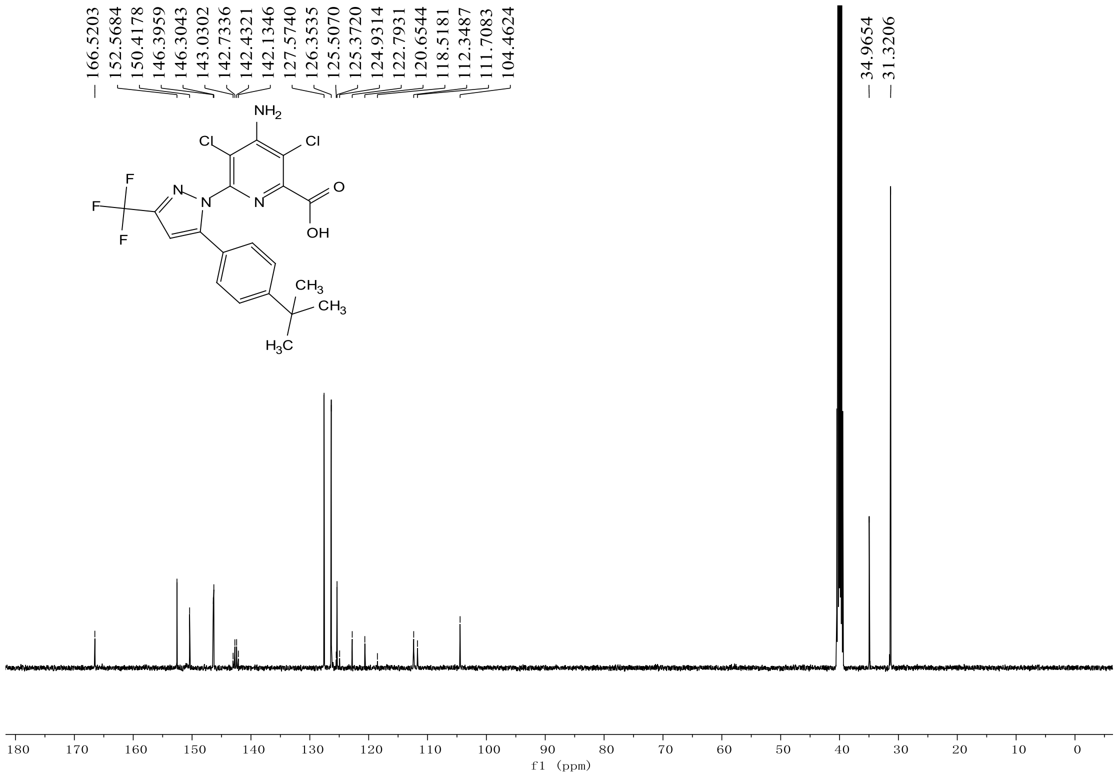

Figure S66. ^{13}C NMR spectrum of compound V-33.
